# Key genes associated with brain metastasis in non-small cell lung cancer: novel insights from bioinformatics analysis

**DOI:** 10.3389/fbinf.2025.1625664

**Published:** 2025-09-18

**Authors:** Shuang Zhao, He Zhang

**Affiliations:** General Hospital of Northern Theater Command of the Chinese People's Liberation Army, Shenyang, China

**Keywords:** brain metastasis, non-small-cell lung cancer, biomarker, signaling pathway, gene

## Abstract

**Background:**

This study aims to investigate potential biomarkers associated with NSCLC-BM and elucidate their regulatory roles in critical pathways involved in cerebral metastatic dissemination.

**Methods:**

The identified DEGs were subjected to functional enrichment analysis. PPI networks were predicted using the STRING database and visualized with Cytoscape. Hub genes were subsequently screened from the PPI network to construct a transcription TF-miRNA regulatory network. Subsequent analyses included: survival analysis, immune infiltration assessment and comprehensive mutational profiling.

**Results:**

Among the 56 identified DEGs, 19 were upregulated while 37 were downregulated. GOntology enrichment analysis revealed significant enrichment in immune response, signaling receptor binding, and extracellular region. KEGG pathway analysis demonstrated predominant involvement in cytokine-cytokine receptor interaction and chemokine signaling pathway. Through Cytoscape-based screening, we identified 10 hub genes: CD19, CD27, IL7R, SELL, CCL5, CCR5, PRF1, GZMK, GZMA, and TIGIT. The TF-miRNA regulatory network analysis uncovered 6 transcription factors (STAT5A/B, NFKB1, EGR1, RELA, and CTCF) and 4 miRNAs(hsa-miR-204, hsa-miR-148b, hsa-miR-618, and hsa-miR-103) as critical transcriptional and post-transcriptional regulators of DEGs.Integrated analyses including Kaplan-Meier survival curves, immune infiltration profiling, and comprehensive mutational analysis demonstrated significant associations with brain metastatic progression in the studied cohort.

**Conclusion:**

This study provides novel biomarkers from a unique perspective for the diagnosis, prognosis, and development of molecular-targeted therapies or immunotherapies for brain metastasis in NSCLC.

## Introduction

**FIGURE 1:**
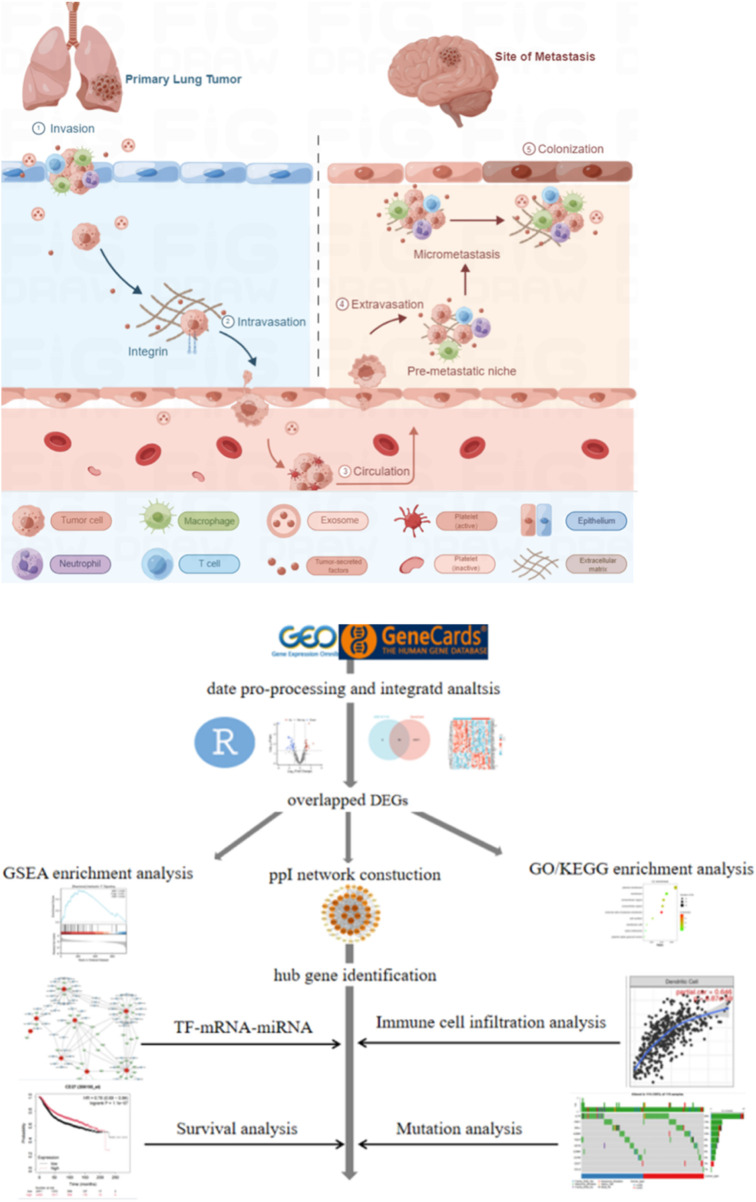
Schematic illustration of brain metastasis in NSCLC and the workflow of bioinformatic analysis.

Lung cancer ranks as the second most commonly diagnosed malignancy worldwide, exceeded only by breast cancer in incidence. It represents the most frequent primary tumor type that metastasizes to the brain, followed by breast cancer and melanoma (Cagney et al., 2017). Non-small cell lung cancer (NSCLC) comprises approximately 85% of all lung cancer cases (Sung et al., 2021; Jonna and Subramaniam, 2019), with brain metastasis (BM) being particularly common in this subgroup. Between 10% and 20% of NSCLC patients present with BM at initial diagnosis (Waqar et al., 2018), and an additional 25%–40% will develop BM throughout the disease course (Page et al., 2020). The prognosis for NSCLC patients with BM remains poor, and symptomatic cases are often associated with rapid deterioration in quality of life (Matsui et al., 2022). Historical reports indicate a median survival of only 4–6 months (Cheng and Perez-Soler, 2018). More recent epidemiological studies show that 15%–20% of NSCLC patients are diagnosed with BM at initial presentation, a figure that increases to 25%–40% over time (Waqar et al., 2018; Nayak et al., 2012; Hubbs et al., 2010). This incidence is even higher among patients with stage IV adenocarcinoma, among whom 40%–50% have BM at diagnosis (Yang et al., 2019). NSCLC patients harboring EGFR or ALK mutations are especially prone to developing BM and exhibit a higher incidence of such events (Gillespie et al., 2023). While historical median survival was reported between several months to one year—and below 6 months without treatment (Ali et al., 2013)-contemporary series report improved outcomes, with a median survival of approximately 15 months in lung adenocarcinoma patients with BM (Sperduto et al., 2020). The Lung-molGPA index further stratifies prognosis, identifying a small subgroup (4%) of patients with scores of 3.5–4.0 who may achieve a median survival of nearly 4 years (Sperduto et al., 2017). This prognostic tool incorporates clinical variables such as age, performance status, number of metastases, and extracranial disease burden, alongside molecular markers including EGFR and ALK mutations (Sperduto et al., 2017). The pathogenesis of BM in NSCLC entails complex crosstalk among tumor cells, immune components, and the specialized brain tumor microenvironment (TME). Metastasis is not solely an intrinsic trait of certain tumors, but a multistep, multidimensional process shaped by mutational landscapes, epigenetic alterations, and growth factor signaling (Srinivasan et al., 2021). As illustrated in Figure 1, the metastatic cascade initiates with local invasion through the basement membrane of the primary lung tumor—a step involving epithelial-mesenchymal transition (EMT) and intravasation into blood or lymphatic vessels. This allows circulating tumor cells (CTCs) to circumvent host immune surveillance and survive in circulation. Nevertheless, the precise mechanisms driving BM remain inadequately characterized, impeding the development of more effective treatment approaches.

## Results

### Identification of differentially expressed genes (DEGs)

**FIGURE 2:**
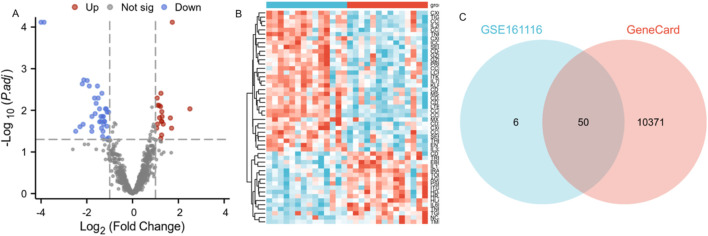
Identification of differentially expressed genes (DEGs) associated with brain metastasis in lung cancer patients. Note: **(A)** Volcano plot of DEGs in GSE161116. X-axis: log2FC; Y-axis: log10 (p-value). Blue: downregulated genes; red: upregulated genes; gray: non-significant genes. **(B)** Heatmap of DEGs in GSE161116. X-axis: samples; Y-axis: genes. Red: high expression; blue: low expression. NSCLC-BM and primary NSCLC samples were clearly separated into two distinct clusters. **(C)** Venn diagram showing overlapping DEGs between GSE161116 and GeneCards databases.

Based on the GSE161116 microarray dataset (GPL19965 platform), this study employed a systematic bioinformatics pipeline for differentially expressed gene (DEG) identification. Data normalization: Raw expression profiles underwent background correction and quantile normalization via the RMA algorithm to eliminate batch effects. Differential analysis: The limma package was applied to identify DEGs between primary NSCLC (n = 14) and NSCLC-BM (n = 14), with thresholds set at |log2FC| >1 and FDR < 0.05, yielding 779 significant DEGs.Visualization:A volcano plot highlighted 56 robust DEGs (19 upregulated, 37 downregulated) (Figure 2A). Venn diagram analysis revealed 50 core overlapping genes between GSE161116 DEGs and the GeneCards BM-related gene set (Figure 2C; Supplementary Table S1). Hierarchical clustering heatmap analysis (pheatmap package) of these 50 intersecting genes demonstrated heterogeneous expression patterns across groups (Figure 2B).

### Enrichment analysis of DEGs

**FIGURE 3:**
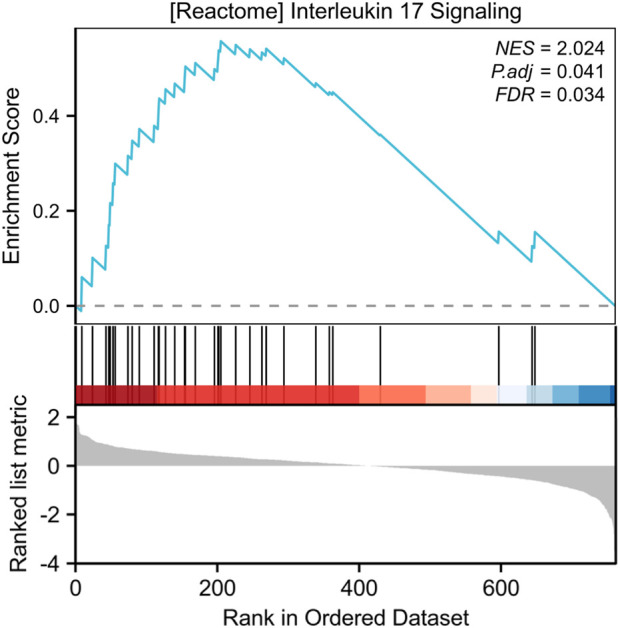
GSEA enrichment analysis results of DEGs between NSCLC and NSCLC-BM groups.

**FIGURE 4:**
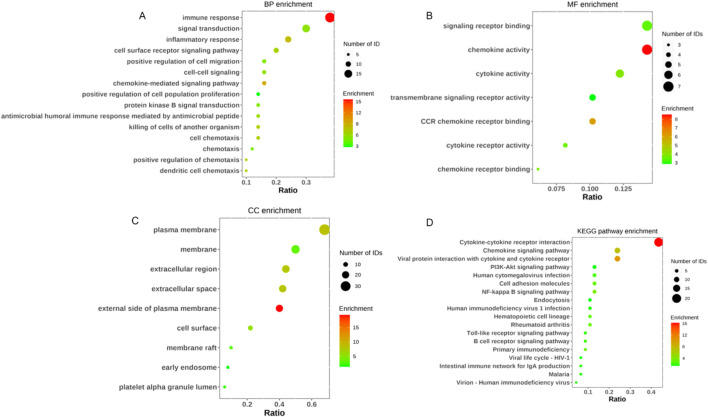
GO enrichment and KEGG pathway analysis of DEGs in NSCLC 和 NSCLC-BM group. **(A)** GO categories of BP. **(B)** GO categories of MF. **(C)** GO categories of CC. **(D)** KEGG pathway analysis of the DEGs.

Our systematic analysis integrating GSEA and multidimensional functional annotation revealed distinct molecular regulatory characteristics of NSCLC brain metastasis (NSCLC-BM). Using the MSigDB database (C2: curated gene sets), we observed marked upregulation of the “interleukin-17 signaling pathway” (NES = 2.024, FDR = 0.024), suggesting that an IL-17-mediated proinflammatory microenvironment may facilitate central nervous system colonization through the TLR/NF-κB axis (Figure 3). To further interpret the functional implications of the differentially expressed genes (DEGs), we performed comprehensive functional enrichment analyses. Detailed results of the GO enrichment analysis are presented in Table1. The most significantly enriched biological process (BP) terms included immune response, signal transduction, inflammatory response, and cell surface receptor signaling pathway (Figure 4A). For molecular function (MF), the top enriched terms were signaling receptor binding, chemokine activity, cytokine activity, and transmembrane signaling receptor activity (Figure 4B). Notably, the key cellular component (CC) terms included plasma membrane, membrane, extracellular region, and extracellular space (Figure C). The KEGG pathway enrichment analysis further highlighted eighteen significantly enriched pathways (Table2), which are visualized in Figure 4D. Major pathways included Cytokine-cytokine receptor interaction, Chemokine signaling pathway, Viral protein interaction with cytokine and cytokine receptor, PI3K-Akt signaling pathway, and Human cytomegalovirus infection.

**TABLE 1:** GO functional enrichment analysis for the DEGs.

Term	*-*log (*P*-value)	Uniprot ID
BP		
immune response	2.60E-16	CCL5/CD48/CCL19/CCR5/IL7R/CD22
signal transduction	3.14E-06	IL2RB/CD48/LTB/IL7R/CCL18
inflammatory response	6.54E-09	CCL5/CCL19/CCR5/CXCL13/CCL18
cell surface receptor signaling pathway	2.02E-07	CCR5/CXCL13/IL7R/IL17RA
positive regulation of cell migration	6.91E-06	CCL5/CCL19/CCL18/THBS1/HRAS
cell-cell signaling	1.78E-06	CCL5/CCR5/CXCL13/CCL18
chemokine-mediated signaling pathway	4.43E-10	CCL5/CCL19/CCR5/CXCL13/CCL18
positive regulation of cell population proliferation	0.001728536	IL7R/THBS1/HRAS/IL6R
protein kinase B signal transduction	9.55E-06	CCL5/CD19/TREM2/CCL19/THBS1
MF		
signaling receptor binding	3.66E-04	TIGIT/CD22/TNFSF13B/IL17RA
chemokine activity	2.91E-09	CCL5/CCL19/CXCL13/CCL18
cytokine activity	1.14E-04	IL32/TGFB2/CXCL9/EBI3/LTB
transmembrane signaling receptor activity	0.001309825	CD79A/KLRB1/CD27/TREM2
CC		
plasma membrane	3.43E-09	CD19/CCR5/TIGIT/SELL/CD27/IL7R
membrane	6.20E-04	PRF1/CCR5/TIGIT/IL17RA/CD22
extracellular region	1.01E-08	PRF1/IL17RA/GZMK/CCL5/CD27/IL7R
extracellular space	7.49E-09	GZMA/GZMK/SELL/CCL5

Category refers to the GO functional categories.

**TABLE 2:** Pathway enrichment analysis for the DEGs.

Term	*-*log (*P*-value)	Uniprot ID
Cytokine-cytokine receptor interaction	6.06E-17	IL17RA/CCL5/IL2RB/CD27/CCR5/IL7R
Chemokine signaling pathway	3.12E-08	CCL5/CXCR4/CCL19/CCR5
Viral protein interaction with cytokine and cytokine receptor	4.85E-11	CCL5/CCL19/CCR5/CCL18/IL6R
PI3K-Akt signaling pathway	0.035395822	CD19/IL2RB/IL7R/THBS1/HRAS/IL6R
Human cytomegalovirus infection	0.005486748	CCL5/CXCR4/CCR5/HRAS/IL6R
Cell adhesion molecules	0.001160078	SELL/NCAM1/TIGIT/CD22

Category refers to the pathway functional categories.

### PPI network construction and hub gene selection

**FIGURE 5:**
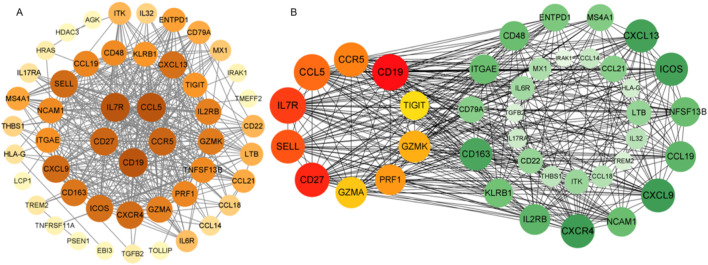
Protein-protein interaction (PPI) network visualized using Cytoscape software. Figure Note: **(A)** Node color intensity corresponds to degree value. **(B)** The network contains 40 nodes and 368 edges, with progressively redder hues indicating higher degree scores as measured by CytoHubba. Hub-DEGs in the PPI network are distinguished by unique coloring, while green nodes represent associated proteins.

We constructed a protein-protein interaction (PPI) network using the STRING database based on the 50 overlapping DEGs and visualized it with Cytoscape to identify highly interconnected hub proteins (hub-DEGs). The PPI network contained 64 nodes and 174 edges (Figure 5A). Among these genes, the top 10 proteins with the highest degree of interaction were identified as key hub genes: CD19, CD27, IL7R, SELL, CCL5, CCR5, PRF1, GZMK, GZMA, and TIGIT (Figure 5B). Literature mining revealed that these candidate genes are predominantly involved in: (1) immune synapse formation (CD27-IL7R axis), (2) T-cell exhaustion (TIGIT-PRF1 pathway), and (3) chemokine-mediated blood-brain barrier penetration (CCL5-CCR5 signaling). These findings suggest their potential role in driving brain metastasis progression through modulation of tumor-immune microenvironment interactions.

### Validation data analysis

**FIGURE 6:**
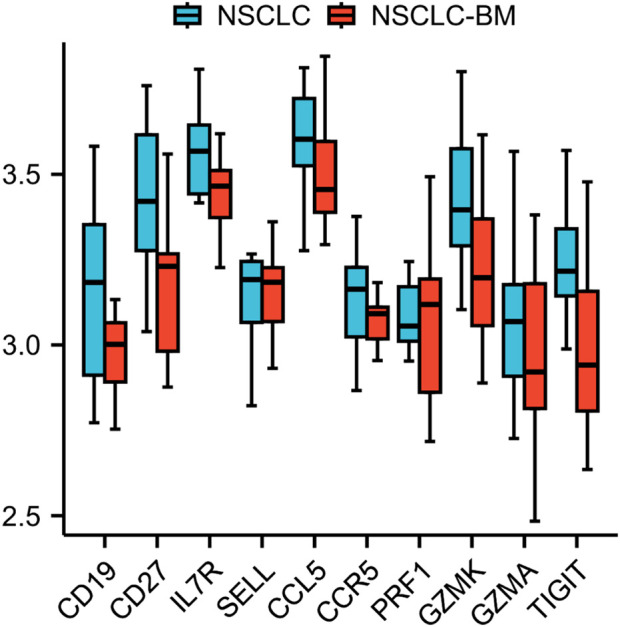
Grouped box plot analysis confirming differential expression.

The ten identified hub genes were validated using additional GEO datasets. The GSE248830 dataset, which includes 11 NSCLC and 11 NSCLC-BM samples, was employed to examine differential expression between primary and metastatic tumors. Preliminary analysis revealed that the expression levels of these ten hub genes were significantly downregulated following metastasis, a trend consistent with previous findings from the GSE161116 dataset. These results further support the reliability of our conclusions. The validation results are presented in Figure 6.

### TF regulatory network analysis of ten genes

**FIGURE 7:**
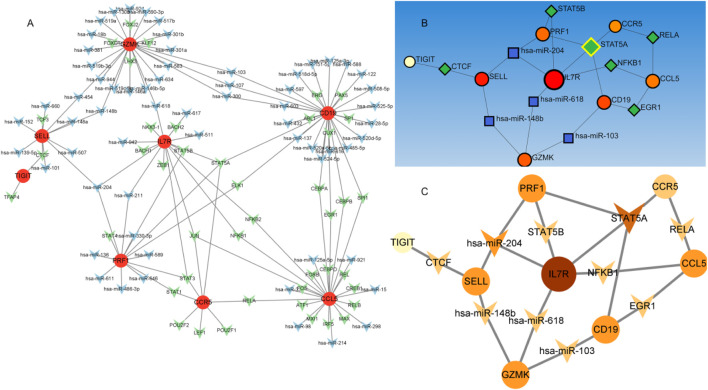
The TF-mRNA -miRNA regulatory network. Figure Note: **(A)** Regulatory network of hub genes. Red circles represent hub genes, green circles denote transcription factors (TFs), and blue circles indicate miRNAs. **(B,C)** Subnetworks of key TF-regulated genes, with node color intensity scaled according to degree values.

We established an integrated TF-mRNA-miRNA regulatory network comprising 10 hub genes, 43 transcription factors (TFs), and 63 miRNAs (Figure 7). Comprehensive analysis of the TF-DEG and miRNA-DEG interaction networks revealed significant regulatory molecules. Notably, 8 of the 10 hub genes were embedded within this regulatory architecture. Subsequent subnetwork analysis identified key transcriptional regulators (STAT5A, STAT5B, NFKB1, EGR1, RELA, and CTCF) and post-transcriptional modulators (hsa-miR-204, hsa-miR-148b, hsa-miR-618, and hsa-miR-103) as pivotal biomolecules governing DEG expression. Mechanistically, 6 TFs (STAT5A/B, NFKB1, EGR1, RELA, and CTCF) emerged as central transcriptional regulators, while the miRNAs exhibited specific target interactions: hsa-miR-204: IL7R and PRF1/SELL; hsa-miR-148b: GZMK and SELL; hsa-miR-618: IL7R and GZMK; hsa-miR-103: CD19 and GZMK; These computational predictions require experimental validation to confirm their biological relevance in NSCLC-BM pathogenesis.

### Survival impact of hub genes in brain metastasis

**FIGURE 8:**
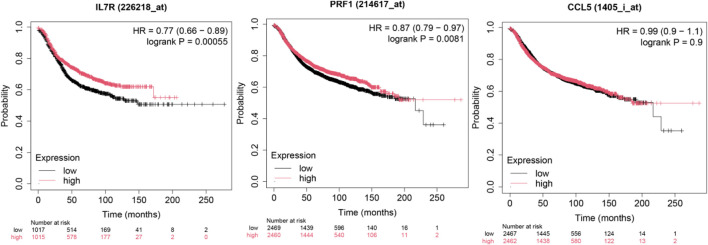
Overall survival analysis of 10 hub DEGs in kmplot website.

To investigate the prognostic significance of the ten hub genes in patients with brain metastasis (BM), we performed Kaplan-Meier survival analysis stratified by median gene expression levels (high vs. low expression groups). The results (Figure 8) demonstrated that decreased expression of these genes was significantly associated with shortened overall survival (OS) in patients with brain metastasis. Notably, prior studies have demonstrated that STAT5A promotes tumor invasion and metastasis by upregulatingCD44 (Szczepanik et al., 2019)—a cancer stem cell (CSC) marker linked to unfavorable prognosis in gastric cancer (GC). Our findings align with this mechanism, suggesting that the identified transcription factors (TFs) and hub genes may collectively accelerate brain metastasis through: Enhanced tumor cell invasiveness (via STAT5A-CD44 axis), Metastatic niche modulation, Post-metastatic transcriptional reprogramming (evidenced by expression downregulation post-metastasis) These results implicate the ten hub genes as critical mediators of lung cancer brain metastasis, potentially governing tumor cell dissemination and survival outcomes. Detailed results are shown in the Supplementary Figure S1.

### Immune cell infiltration analysis

**FIGURE 9:**
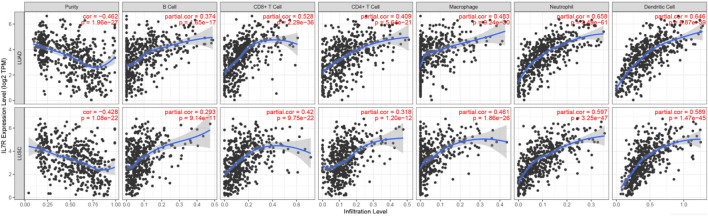
The correlation of IL7R with tumor purity and immune cells in the immune system shows the purity-corrected partial Spearman’s rho value and statistical significance. Log2 (TPM) is the log2 of the Transcript Count Per Million.

To elucidate the relationship between the 10 hub genes and immune cell activity, we performed tumor-infiltrating immune cell (TIIC) profiling. Compositional analysis revealed significant positive correlations between the expression of hub genes (IL7R,PRF1, etc.) and activated immune subsets, including: Memory B cells, Activated CD4+T cells (correlation with IL7R:r = 0.409), CD8+T cells, Notably,IL7R exhibited the strongest associations: B cell activity (r = 0.374, Figure 9), CD4+T cell recruitment (r = 0.409). Additional hub gene–immune interactions are detailed in Supplementary Figure S2. These findings underscore the pivotal role of these genes in modulating B and T cell crosstalk within the tumor microenvironment (TME) of LUAD patients, suggesting their potential as immunomodulatory targets.

### Mutation analysis of 10 crucial genes

**FIGURE 10:**
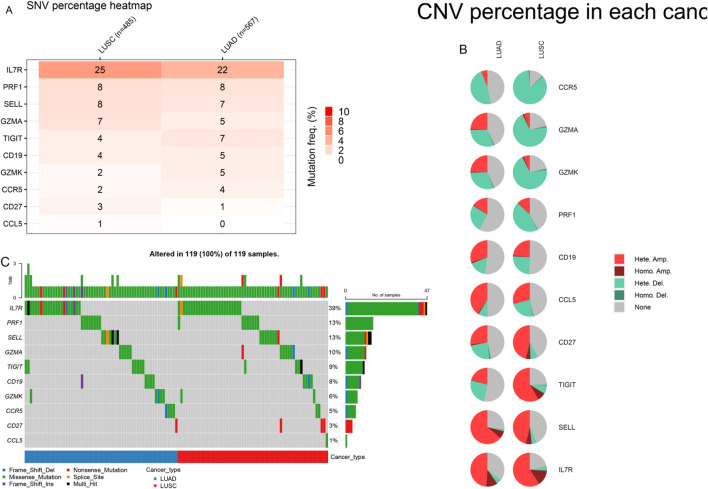
Variant frequency and mutation type of 10 genes in LUAD and LUSC. **(A)** The mutation rate of genes. **(B)** The CNV types of genes. **(C)** The mutation type of genes.

We examined the mutation frequency and mutation types of these 10 hub genes in the GSCA database. The results revealed that IL7R exhibited the highest mutation frequency, followed by PRF1, with missense mutations accounting for 39% and 13% of the alterations in these genes, respectively. Additionally, copy number variation (CNV) analysis demonstrated that IL7R had the highest Figure 10. Notably, prior studies have reported that missense mutations in the perforin (PRF1) gene contribute to hereditary cancer predisposition (Chaudhry et al., 2016). Our findings suggest that mutations in these genes may play a role in cancer brain metastasis, potentially influencing tumor progression and metastatic potential.

## Discussion

The identification of biomarkers associated with lung cancer brain metastasis may provide deeper insights into the molecular mechanisms underlying metastatic progression. This study aimed to analyze NSCLC gene expression data to identify differentially expressed genes (DEGs), elucidate key molecular pathways, determine critical hub proteins, and characterize relevant regulatory biomolecules through a multi-omics data integration framework, with the ultimate goal of discovering potential therapeutic targets for NSCLC. Our gene expression profiling identified 56 DEGs, including 19 upregulated and 37 downregulated genes. Functional enrichment analysis revealed that these DEGs were significantly associated with several oncogenic molecular functions and pathways. GSEA results demonstrated marked upregulation of the “interleukin-17 signaling pathway.” Notably, the interleukin-17 (IL-17) signaling pathway has been previously established to contribute to the progression of lung cancer bone metastasis (Zhou et al., 2023). GO and KEGG enrichment analyses identified several critical biological processes and pathways, including immune response, signal transduction, inflammatory response, cell surface receptor signaling pathway, positive regulation of cell migration, cell-cell signaling, signaling receptor binding, chemokine activity, cytokine activity, transmembrane signaling receptor activity, plasma membrane, membrane, extracellular region, extracellular space, cytokine-cytokine receptor interaction, chemokine signaling pathway, viral protein interaction with cytokine and cytokine receptor, and PI3K-Akt signaling pathway. Existing evidence suggests that inflammatory chemokines and their receptors regulate tumor cell migration and participate in tumor growth, metastasis, angiogenesis, and invasion through interactions between mesenchymal and tumor cells (Cheng et al., 2016; Zhao et al., 2019). All these functions and pathways are significantly associated with cancer development and play crucial roles in the NSCLC microenvironment. Protein-protein interaction (PPI) network analysis has emerged as a promising approach for investigating the fundamental mechanisms of brain metastasis in lung cancer (Sevimoglu and Arga, 2014). Our PPI network analysis revealed hub proteins encoded by hub DEGs. The CCR5/CCL5 signaling axis has been shown to increase infiltration of regulatory T cells (Tregs) and myeloid-derived suppressor cells (MDSCs) into the tumor microenvironment (TME), creating an immune effector cell desert that promotes cancer survival and progression (Sevimoglu and Arga, 2014), while potentially contributing to immunotherapy resistance. This pathway has also demonstrated prognostic and predictive value in metastatic colorectal cancer (CRC) (Suarez-Carmona et al., 2019; Schlechter and Stebbing, 2024). CD27, a member of the TNF receptor superfamily, is essential for T cell immunity generation and long-term maintenance; Pagès et al. (Pages et al., 2005) found that CD27 expression correlates with early metastasis in colorectal cancer. IL-7R has emerged as a potential prognostic marker in breast cancer patients, particularly in maintaining immunologically active states in the TME and promoting immune reconstitution (Yu et al., 2024). PRF1, a crucial cytotoxic molecule, plays a vital role in the killing functions of natural killer (NK) cells and cytotoxic T lymphocytes (CTLs) (Guan et al., 2024). GZMA, GZMK, and PRF1 (Tibbs and Cao, 2022; Paczek et al., 2022; Park et al., 2021; Lavergne et al., 2021) not only induce apoptosis and modulate immune responses within the TME but also exhibit other distinct functions. Inhibition of tumor growth has been associated with reduced expression of the immune checkpoint molecule TIGIT (Shaw et al., 2022). The subnetwork modules containing these hub genes provide strong evidence supporting their reliability as therapeutic targets. The TF–mRNA–miRNA regulatory network analysis identified six transcription factors (STAT5A, STAT5B, NFKB1, EGR1, RELA, and CTCF) and four miRNAs (hsa-miR-204, hsa-miR-148b, hsa-miR-618, and hsa-miR-103) as key transcriptional and post-transcriptional regulators of hub DEGs. Previous studies have reported that various tumor-associated genes are regulated by STAT5A/STAT5B, which maintain multiple cancer-related pathways (Erdogan et al., 2022). NFKB1 plays critical roles in tumor cell invasion and metastasis, with its expression linked to invasion and metastasis across various cancer types (Zhang et al., 2023). EGR1 acts as a pro-metastatic factor in pancreatic cancer cells, promoting cell migration and invasion through the SNAI2–EMT pathway (Wang et al., 2023). Currently, there are limited reports on the tumor-related effects of RELA and CTCF. hsa-miR-204 has been reported to be downregulated and function as a tumor suppressor in various cancers including colorectal cancer, papillary thyroid carcinoma, malignant melanoma, and hepatocellular carcinoma (Chu et al., 2018). Studies have demonstrated significant enrichment of hsa-miR-148b in cancer-related pathways including Wnt, MAPK, and Jak-STAT signaling pathways (Luo et al., 2015). Dysregulation of hsa-miR-618 has been associated with numerous cancers (Radanova et al., 2021). hsa-miR-103a can function as either a tumor promoter or suppressor, modulating tumor progression in various cancers (Li et al., 2021). Survival curve analysis clearly demonstrated significant differences in hub gene expression between high-risk and low-risk groups, indicating their important roles in patient survival. Furthermore, immune infiltration analysis revealed interactions between these key genes and B/T cells in LUAD patients, suggesting their influence on the TME. On the other hand, in cancer cells, gene mutations, amplifications, and deletions can lead to altered target proteins that fail to bind drugs, resulting in drug resistance. In particular, missense mutations may significantly affect protein function (Chen et al., 2004). Our mutation analysis of 10 genes in NSCLC revealed that IL7R and PRF1 had the highest missense mutation rates at 39% and 13%, respectively. It has been demonstrated that IL7R mutations activate downstream IL7R signaling independent of IL7 and promote cell transformation and tumor formation, indicating that IL7R exon 6 mutations are gain-of-function mutations (Kim et al., 2013). PRF1 plays important roles in various aspects of tumor cell development, immune escape mechanisms, cancer immunotherapy, and prognosis (Guan et al., 2024). However, the functional characteristics of most missense mutations in IL7R and PRF1 in tumors remain poorly characterized. Importantly, we found strong associations between IL7R/PRF1 mutation co-occurrence and immune-related pathways, particularly B cell and T cell signaling. The presence of infiltrating immune cells in these tumors, some of which inhibit or promote disease progression, further supports the involvement of immune pathways. In conclusion, although this study has certain limitations including a small sample size and lack of clinical sample validation, our current analysis has identified several key genes and pathways closely associated with NSCLC-BM that may enhance current understanding of its complex mechanisms. Notably, these findings warrant further investigation and experimental validation.

## Conclusion

This study employed bioinformatics approaches to compare non-small cell lung cancer (NSCLC) and brain metastasis (NSCLC-BM) samples, leading to the preliminary identification of 56 differentially expressed genes (DEGs) potentially associated with metastatic progression. These genes were significantly enriched in key pathways including cytokine-cytokine receptor interaction, chemokine signaling pathway, viral protein-cytokine receptor interaction, and the PI3K-Akt signaling pathway. Among them, ten genes—CD19, CD27, IL7R, SELL, CCL5, CCR5, PRF1, GZMK, GZMA, and TIGIT—were selected as potential hub genes. Furthermore, predictions suggested that miRNAs such as hsa-miR-204 and hsa-miR-148b, along with certain transcription factors, may contribute to metastasis by modulating the tumor immune microenvironment. It is important to emphasize that the findings of this study are computational predictions, and all identified genes and regulatory molecules remain candidate biomarkers that have not been experimentally validated. None of the conclusions presented should be interpreted as established biomarkers or clinically applicable outcomes. These predictions require further experimental validation—including qRT-PCR, immunohistochemistry, independent patient cohort analyses, and *in vitro*/*in vivo* functional assays—to confirm their biological significance and translational potential. Future research will focus on experimental verification to evaluate the diagnostic or therapeutic value of these candidate molecules.

## Methods

### Microarray data

The GSE161116 dataset was obtained from the NCBI GEO database (https://www.ncbi.nlm.nih.gov/geo/; GPL19965 platform). This study included 28NSCLC patients with brain metastasis (BM) who underwent surgery at Seoul National University Hospital (SNUH) between January 2013 and March 2018. Clinicopathological data—including age, sex, smoking history, tumor genetic status, treatment, and follow-up records—were retrieved from electronic medical records. Pathological staging was based on the 8th edition of the AJCC staging system (Song et al., 2021). Additionally, GeneCards (Stelzer et al., 2016) was searched using the keyword “lung cancer brain metastasis” to extract relevant target genes.

### Data processing

Differentially expressed genes (DEGs) between primary NSCLC and NSCLC-BM were identified using the limma package (Suzuki et al., 2019) in R (version 4.2.1),with thresholds set at |logFC| > 1 and P < 0.05 (NSCLC without BM vs. NSCLC with BM). The GSE161116 dataset contained 14 NSCLC and 14 NSCLC-BM samples. Overlapping DEGs between GSE161116 and GeneCards (March 2025) were visualized using ggplot2 (Zeng et al., 2022) and VennDiagram packages in R.

### DEG enrichment analysis

Functional enrichment analysis was performed using:Gene Set Enrichment Analysis (GSEA) with the MSigDB Collections (https://www.gsea-msigdb.org/gsea/msigdb/index.jsp) as the reference gene set (500 permutations, significance threshold: p. adj<0.25). Results were visualized via ggplot2 (Zeng et al., 2022). DAVID (v6.8; http://david.ncifcrf.gov) for Gene Ontology (GO) and KEGG pathway analysis. GO terms included biological processes (BP), cellular components (CC), and molecular functions (MF). P < 0.05 was considered statistically significant.

### PPI network construction and hub gene identification

Protein-protein interaction (PPI) networks were built using STRING (v10.0; http://string-db.org; interaction score cutoff: 0.4) and visualized in Cytoscape (v3.9.1). Hub genes were identified via CytoHubba and MCODE plugins, applying 12 ranking algorithms to select the top 10 nodes per method.

### Hub gene-TF-miRNA regulatory network

A mRNA-TF-miRNA co-regulatory network was constructed using NetworkAnalyst (Xia et al., 2015), integrating data from TF-miRNA interaction databases, and visualized in Cytoscape.

### Survival analysis

The Kaplan-Meier Plotter (http://kmplot.com/analysis/) assessed the impact of hub genes on overall survival (OS). Patients were stratified into high- and low-expression groups based on median expression levels (Tang et al., 2019). Differences were evaluated via log-rank test (P < 0.05).

### Immune infiltration analysis

The TIMER 2.0 platform (https://cistrome.shinyapps.io/timer/) analyzed correlations between hub gene expression and immune cell abundance using purity-adjusted Spearman’s rank correlation.

### Mutation analysis

Genomic alterations (mutations and copy number variations, CNVs) in hub genes were analyzed via GSCA (http://gsca.bio-data.cn) to determine mutation frequencies and functional impacts.

### Statistical analysis

To account for multiple hypothesis testing in the identification of differentially expressed genes, the false discovery rate (FDR) was controlled using the Benjamini–Hochberg procedure. The statistical thresholds were set at |log_2_FC|>1 and adjusted p-value (FDR) < 0.05.

## Data Availability

The original contributions presented in the study are included in the article/Supplementary Material, further inquiries can be directed to the corresponding author.
