# Supplementary figures and images for: Key genes associated with brain metastasis in non-small cell lung cancer: novel insights from bioinformatics analysis
Source: Front Bioinform. 2025 Sep 18;5:1625664. doi: 10.3389/fbinf.2025.1625664 (PMC12488587; doi:10.3389/fbinf.2025.1625664)

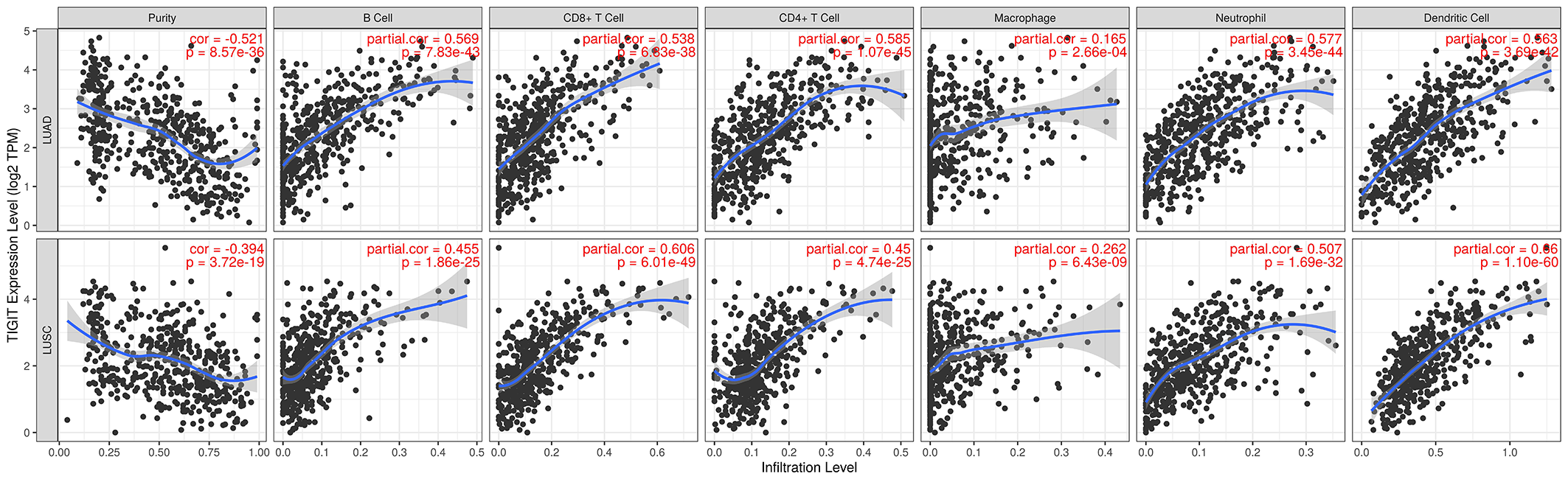

Supplement: Supplementary file 1 [file Image14.png]

# GZMK (206666\_at)

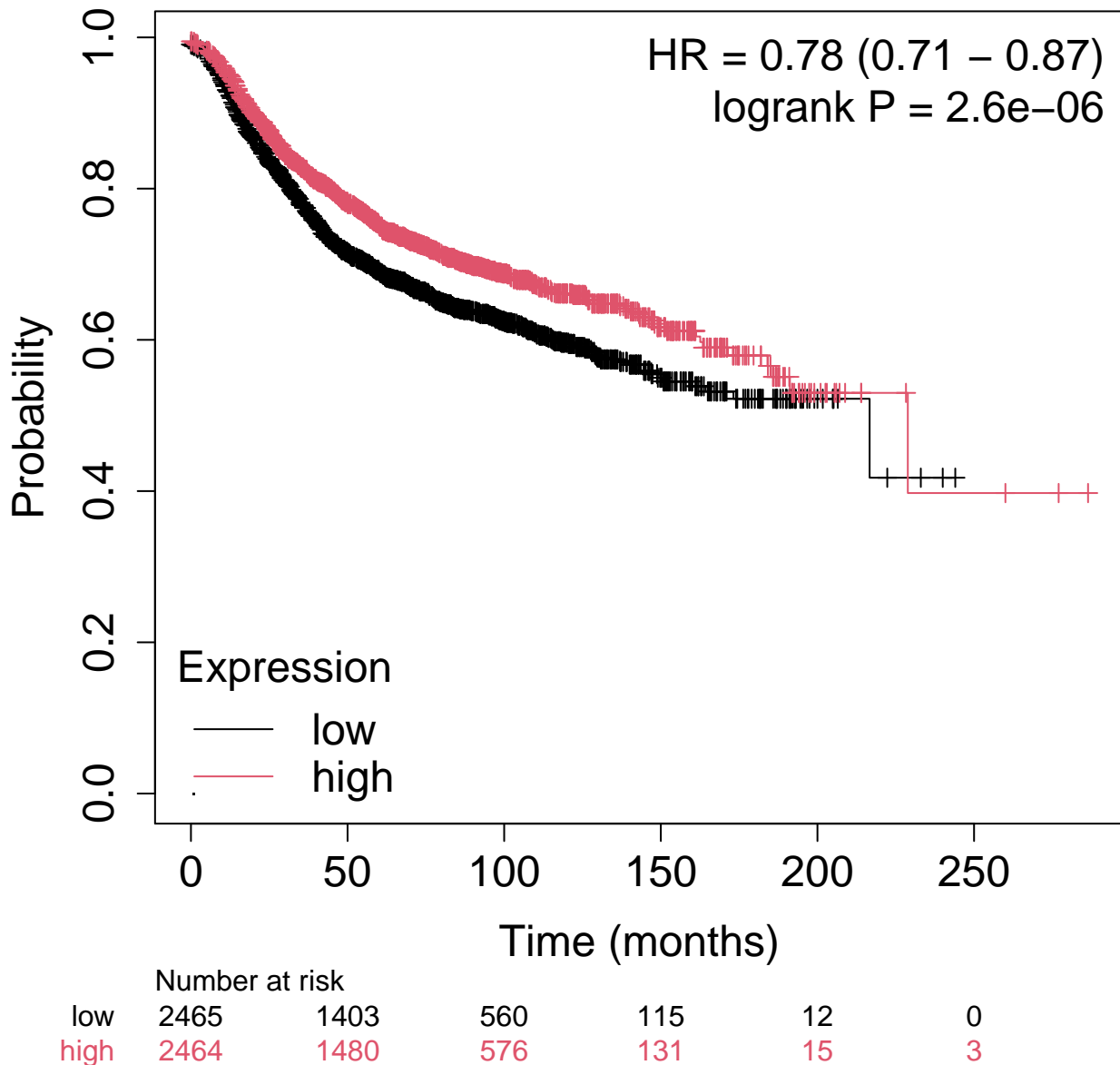

Supplement: Supplementary file 2 [file DataSheet7.pdf]

# CCL5 (1405\_i\_at)

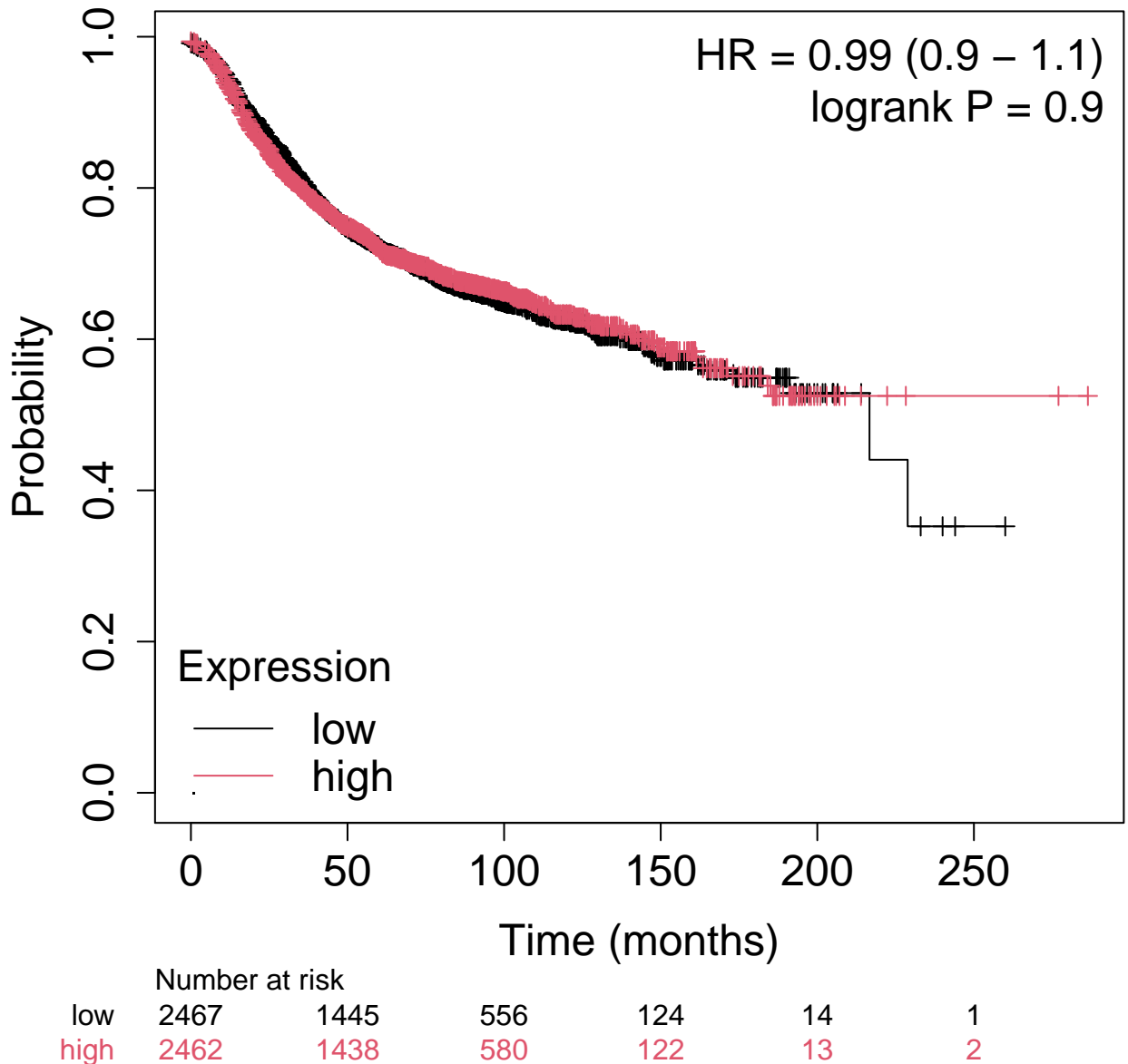

Supplement: Supplementary file 3 [file DataSheet2.pdf]

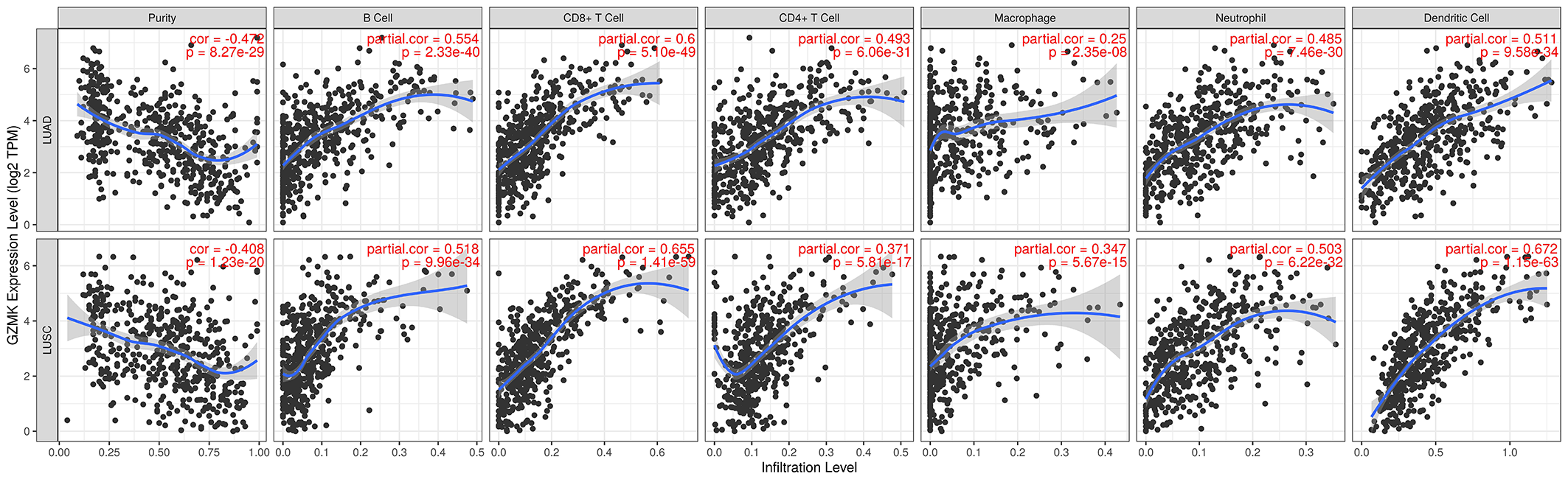

Supplement: Supplementary file 4 [file Image11.png]

# CD19 (206398\_s\_at)

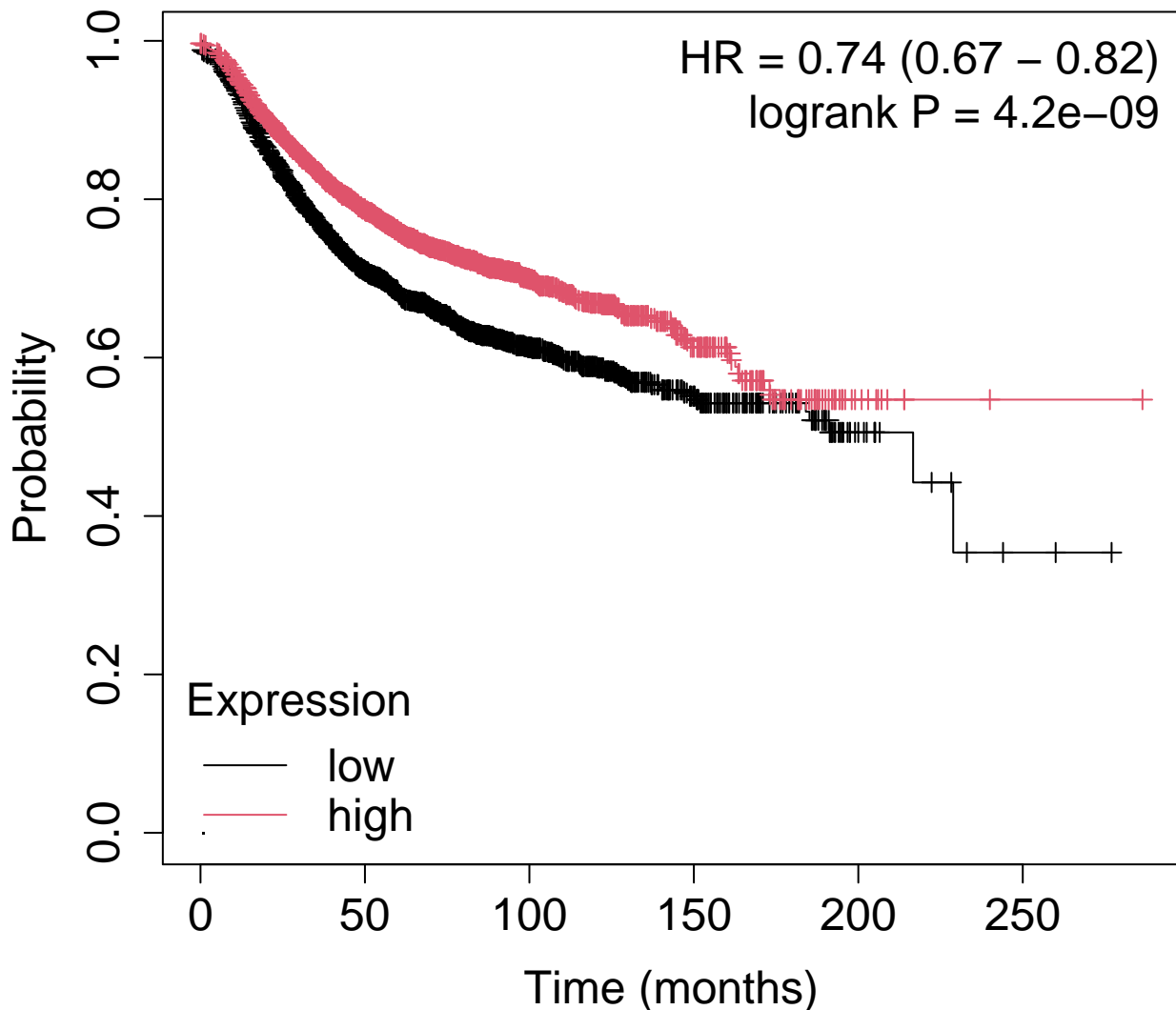

Number at risk

|      |      |      |     |     |    |   |
|------|------|------|-----|-----|----|---|
| low  | 2474 | 1380 | 522 | 133 | 16 | 2 |
| high | 2455 | 1503 | 614 | 113 | 11 | 1 |

Supplement: Supplementary file 5 [file DataSheet4.pdf]

# CTLA3 (205488\_at)

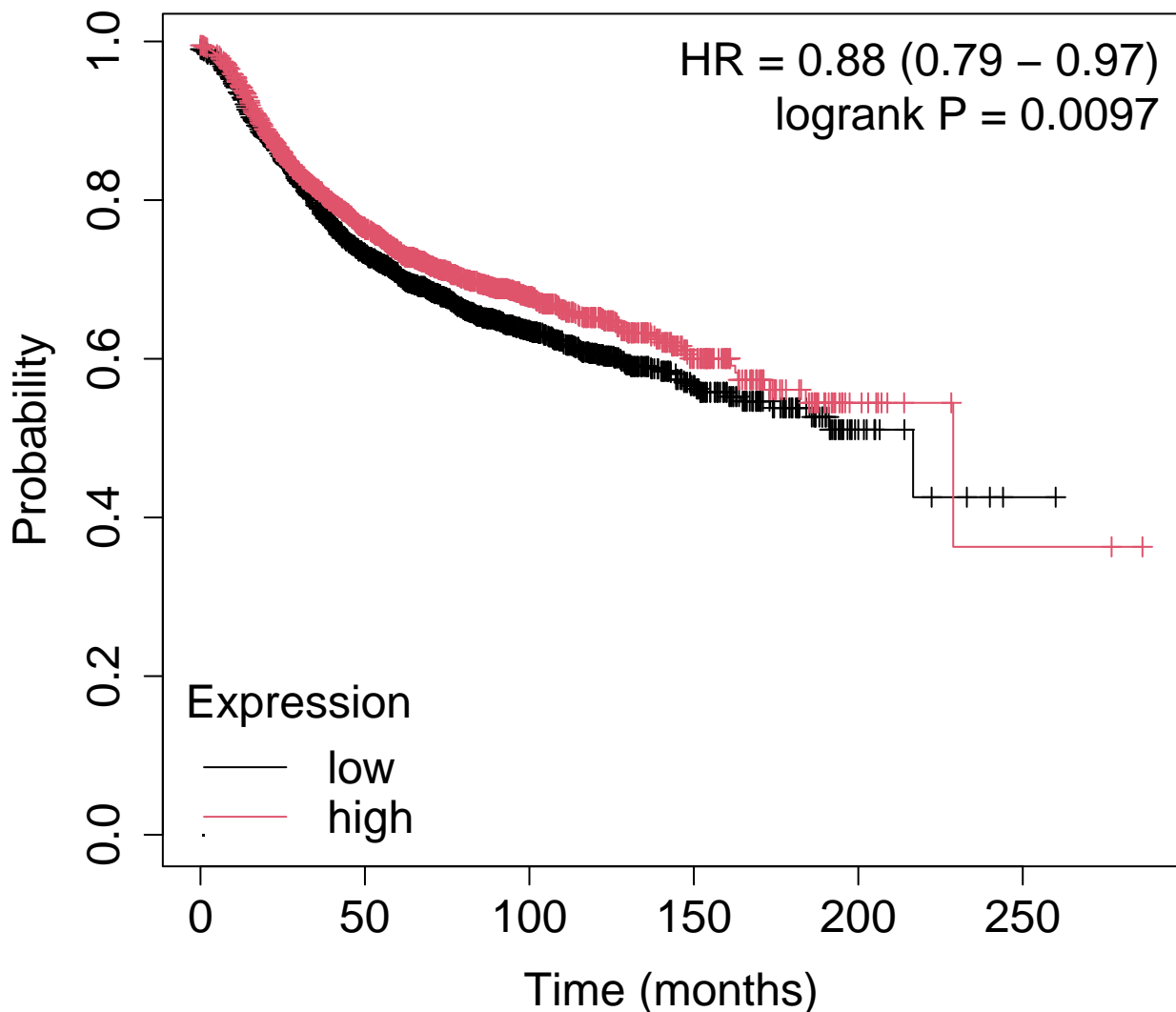

Number at risk

|      |      |      |     |     |    |   |
|------|------|------|-----|-----|----|---|
| low  | 2470 | 1469 | 586 | 140 | 15 | 1 |
| high | 2459 | 1414 | 550 | 106 | 12 | 2 |

Supplement: Supplementary file 6 [file DataSheet6.pdf]

# PRF1 (214617\_at)

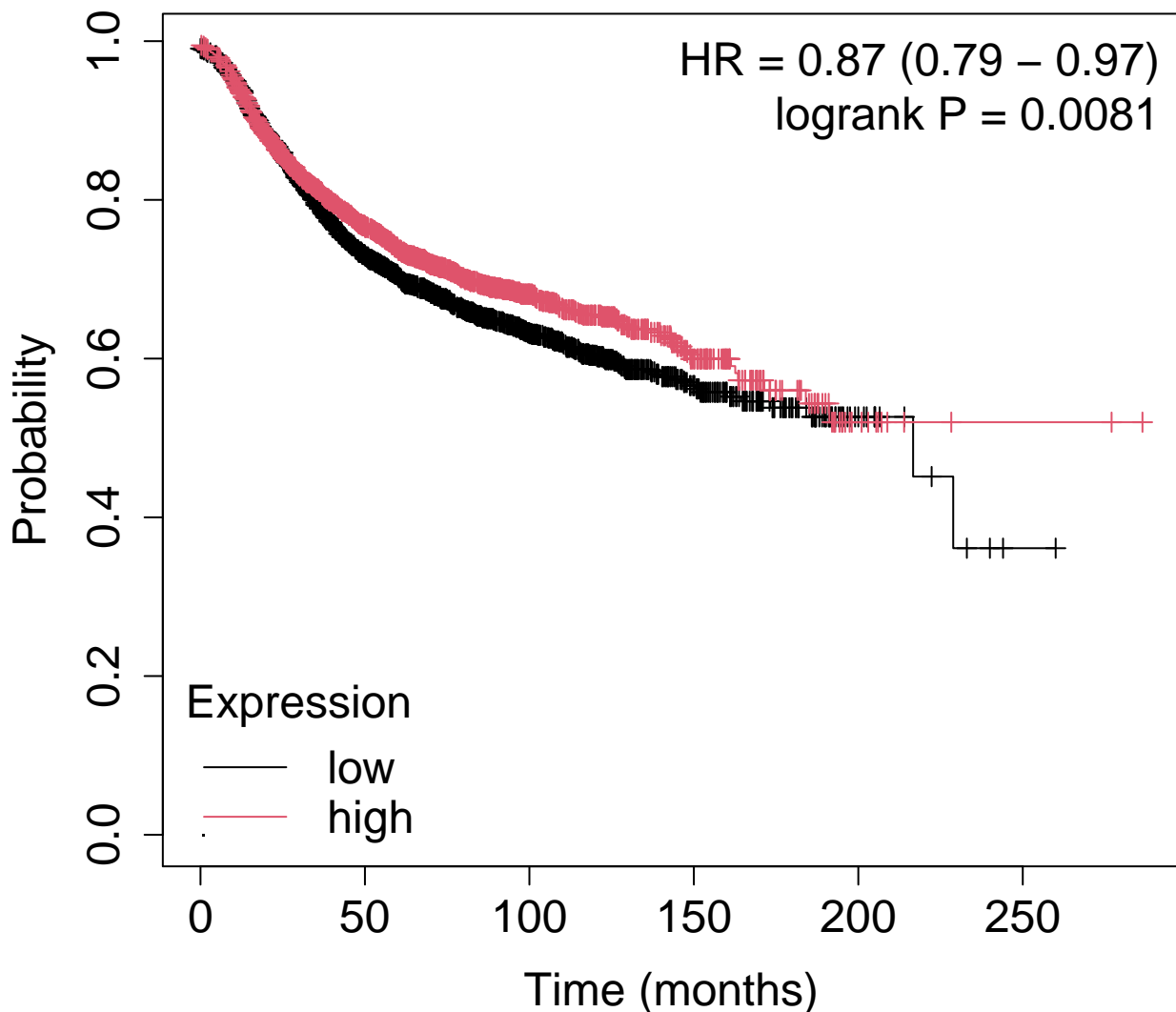

Number at risk

|      |      |      |     |     |    |   |
|------|------|------|-----|-----|----|---|
| low  | 2469 | 1439 | 596 | 140 | 16 | 1 |
| high | 2460 | 1444 | 540 | 106 | 11 | 2 |

Supplement: Supplementary file 7 [file DataSheet9.pdf]

# TIGIT (240070\_at)

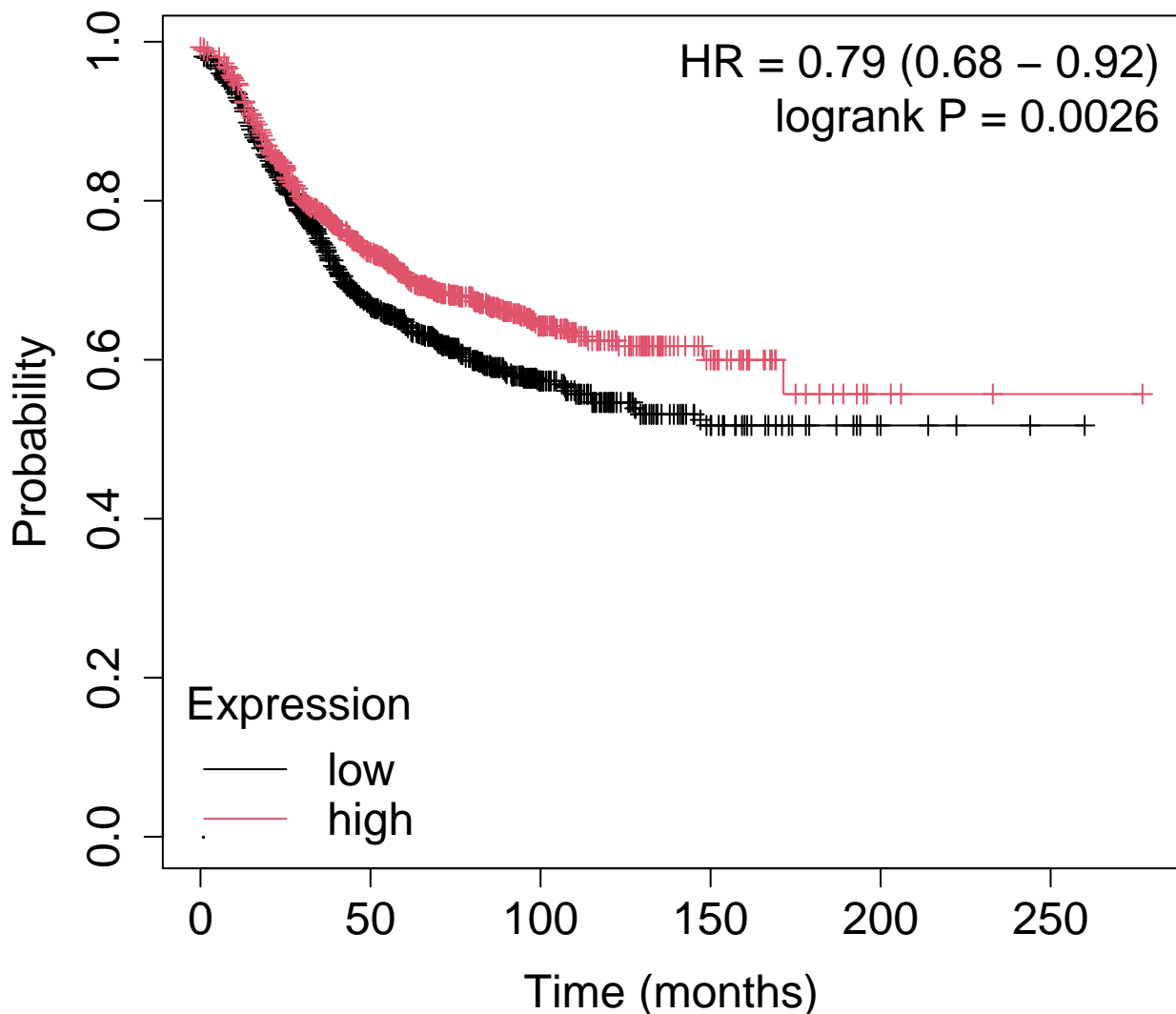

Number at risk

|      |      |     |     |    |   |   |
|------|------|-----|-----|----|---|---|
| low  | 1018 | 518 | 158 | 35 | 6 | 1 |
| high | 1014 | 574 | 188 | 33 | 4 | 1 |

Supplement: Supplementary file 8 [file DataSheet11.pdf]

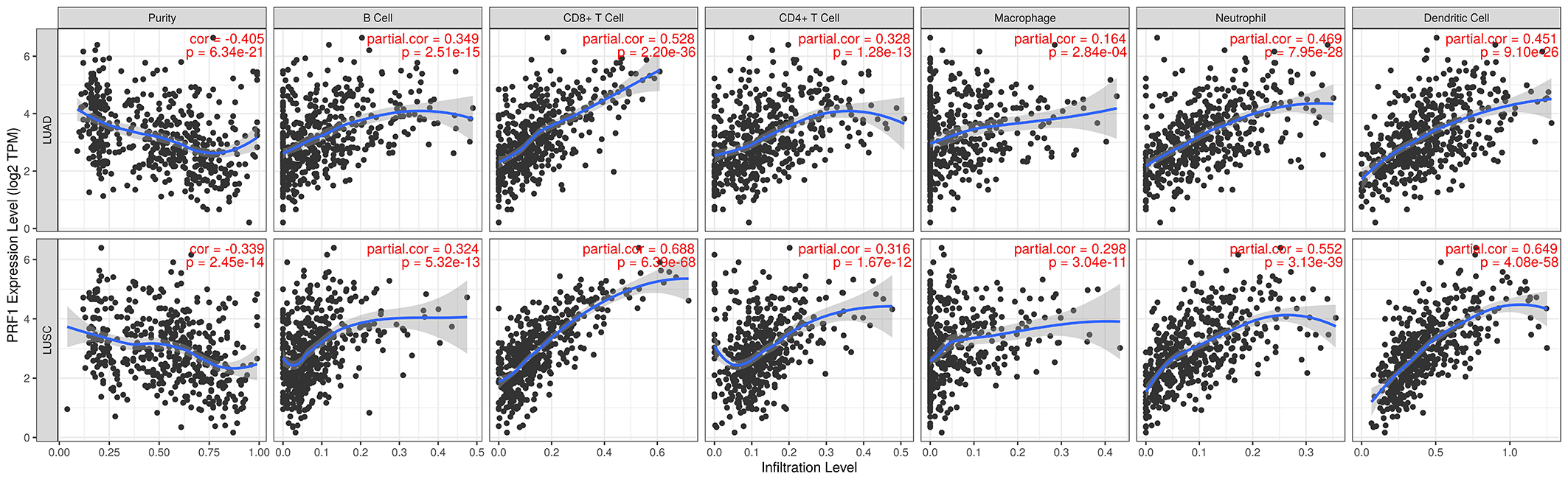

Supplement: Supplementary file 9 [file Image12.png]

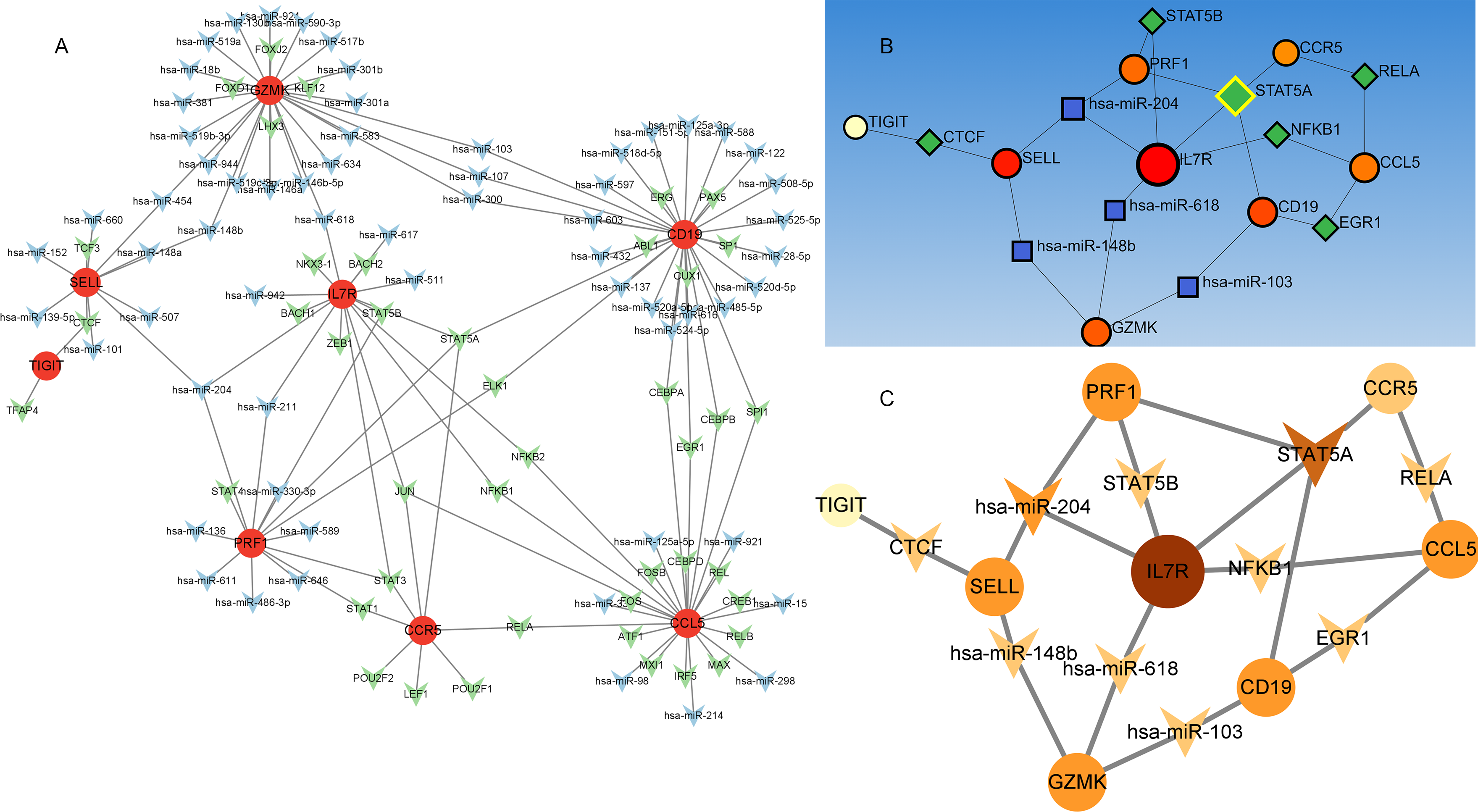

Supplement: Supplementary file 10 [file Image5.png]

# CD195 (206991\_s\_at)

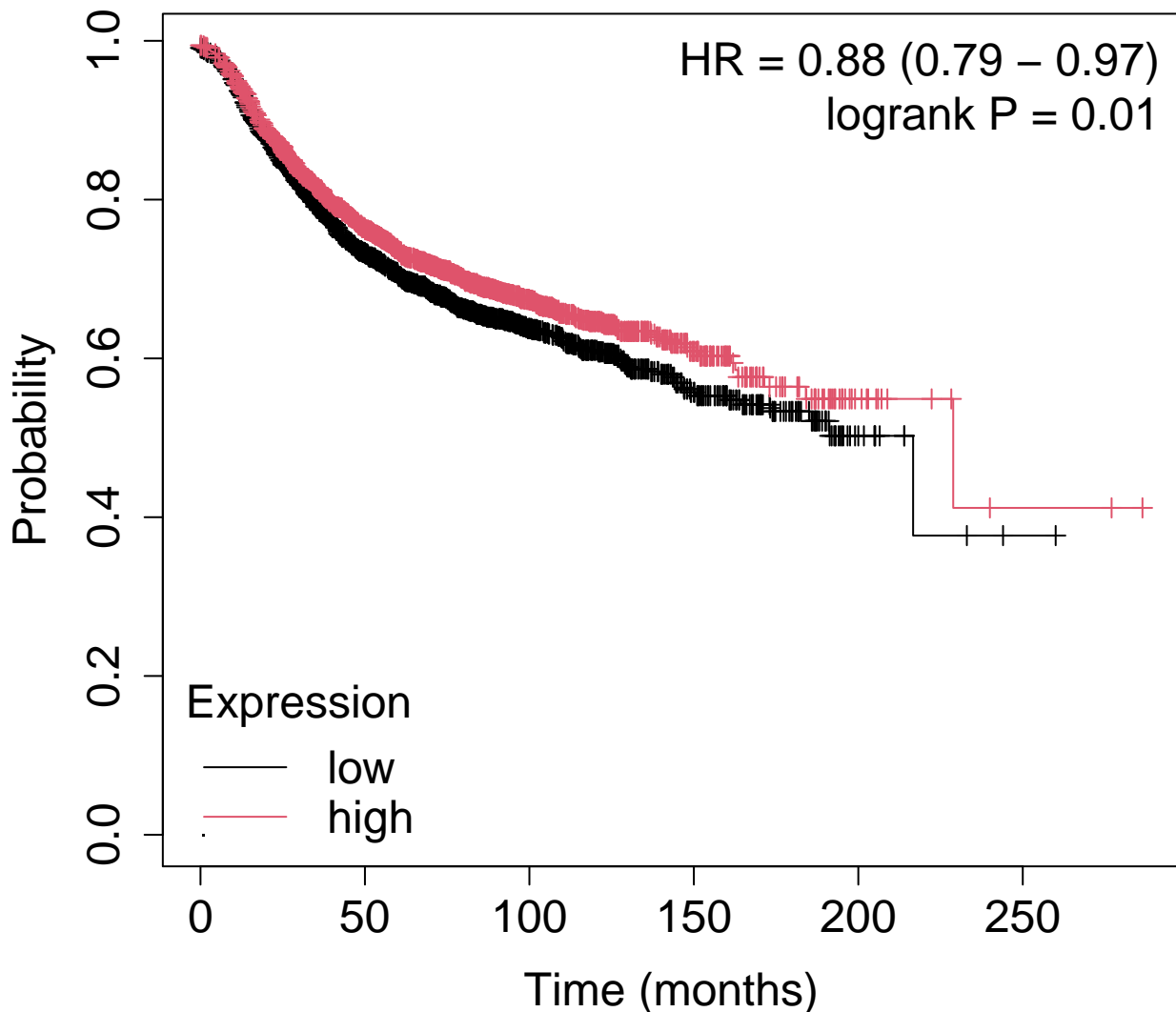

Number at risk

|      |      |      |     |     |    |   |
|------|------|------|-----|-----|----|---|
| low  | 2467 | 1396 | 513 | 134 | 13 | 1 |
| high | 2462 | 1487 | 623 | 112 | 14 | 2 |

Supplement: Supplementary file 11 [file DataSheet3.pdf]

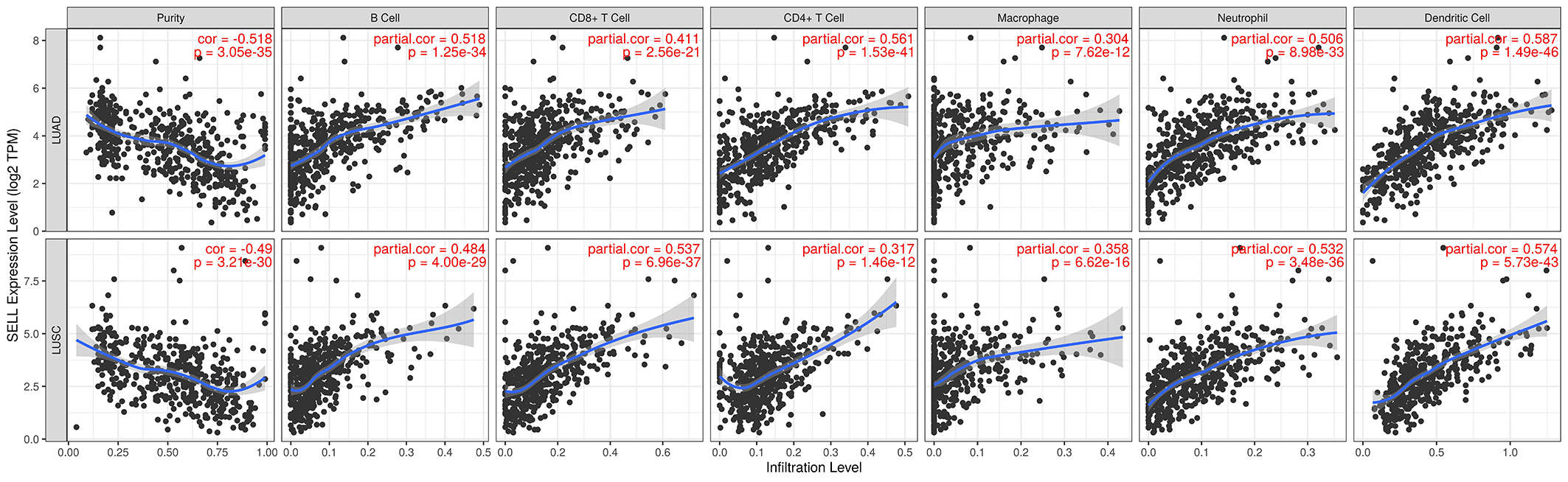

Supplement: Supplementary file 12 [file Image13.png]

# CD27 (206150\_at)

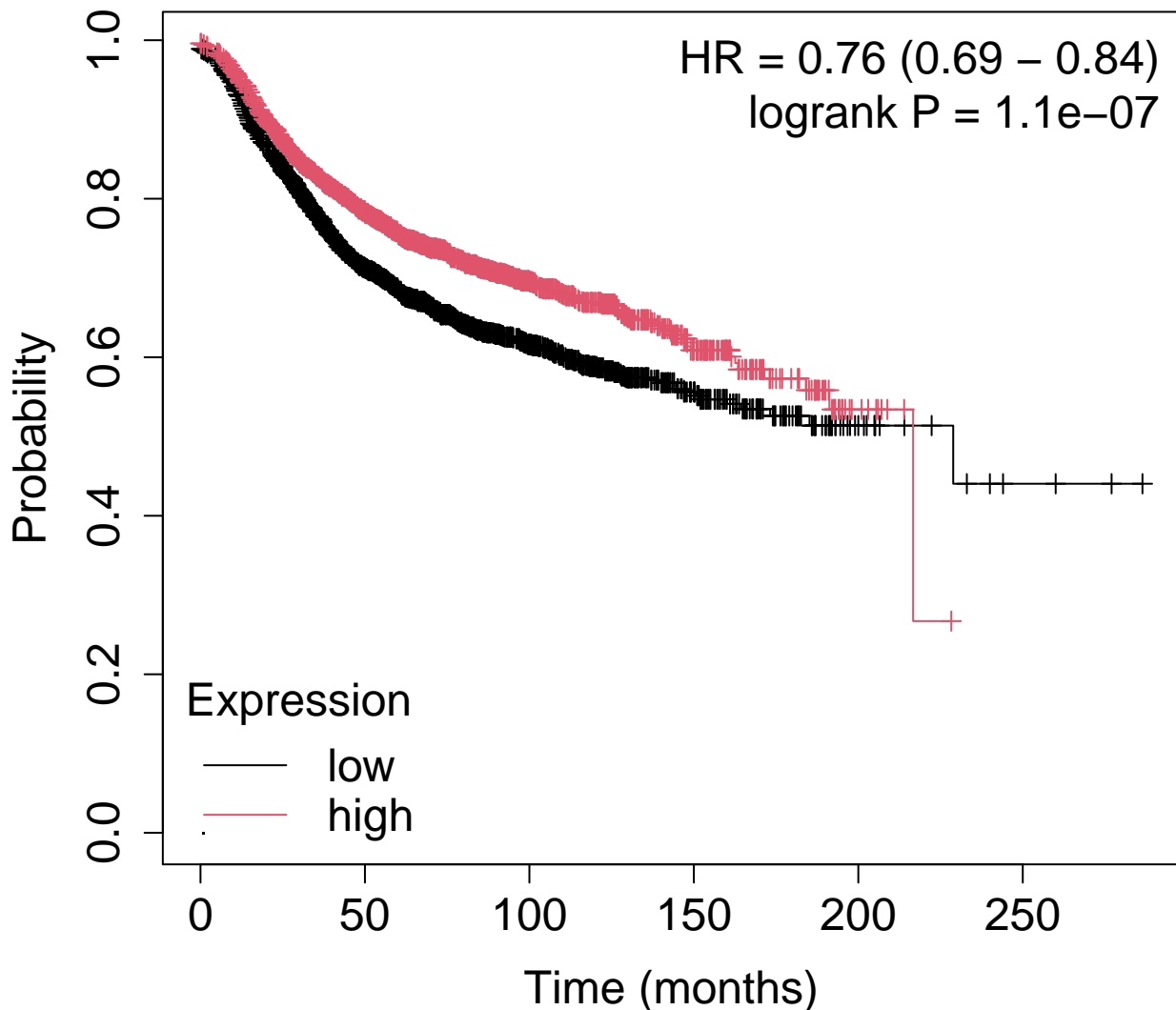

Number at risk

|      |      |      |     |     |    |   |
|------|------|------|-----|-----|----|---|
| low  | 2471 | 1372 | 568 | 127 | 17 | 3 |
| high | 2458 | 1511 | 568 | 119 | 10 | 0 |

Supplement: Supplementary file 13 [file DataSheet5.pdf]

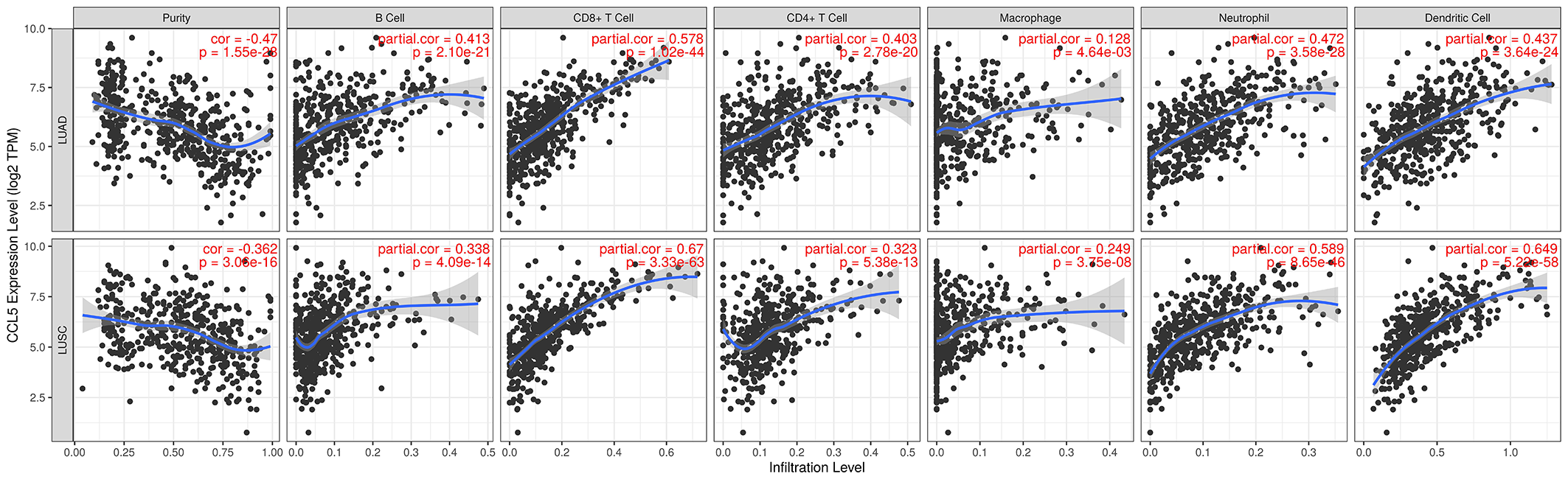

Supplement: Supplementary file 14 [file Image7.png]

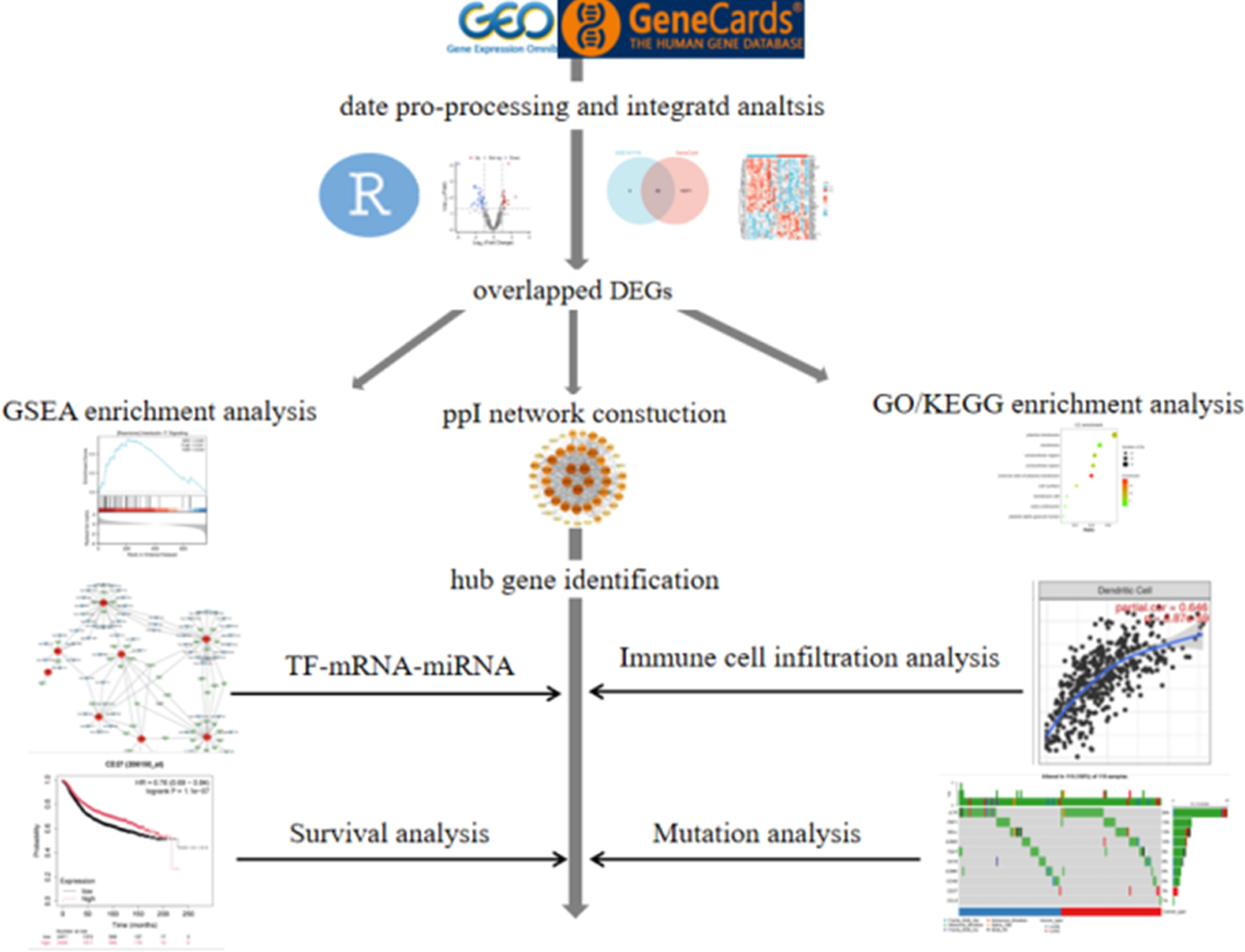

Supplement: Supplementary file 15 [file Image2.png]

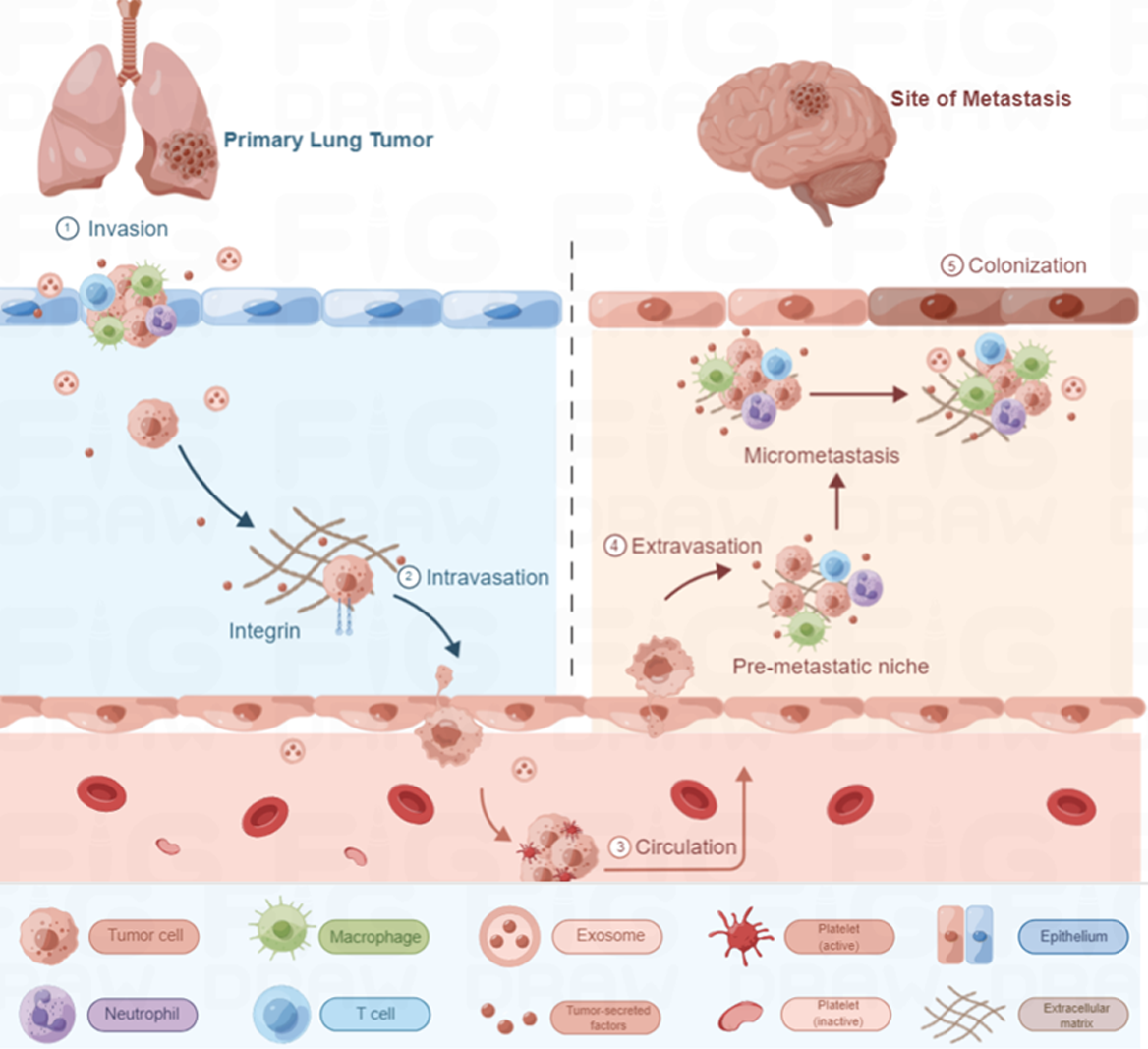

Supplement: Supplementary file 16 [file Image1.png]

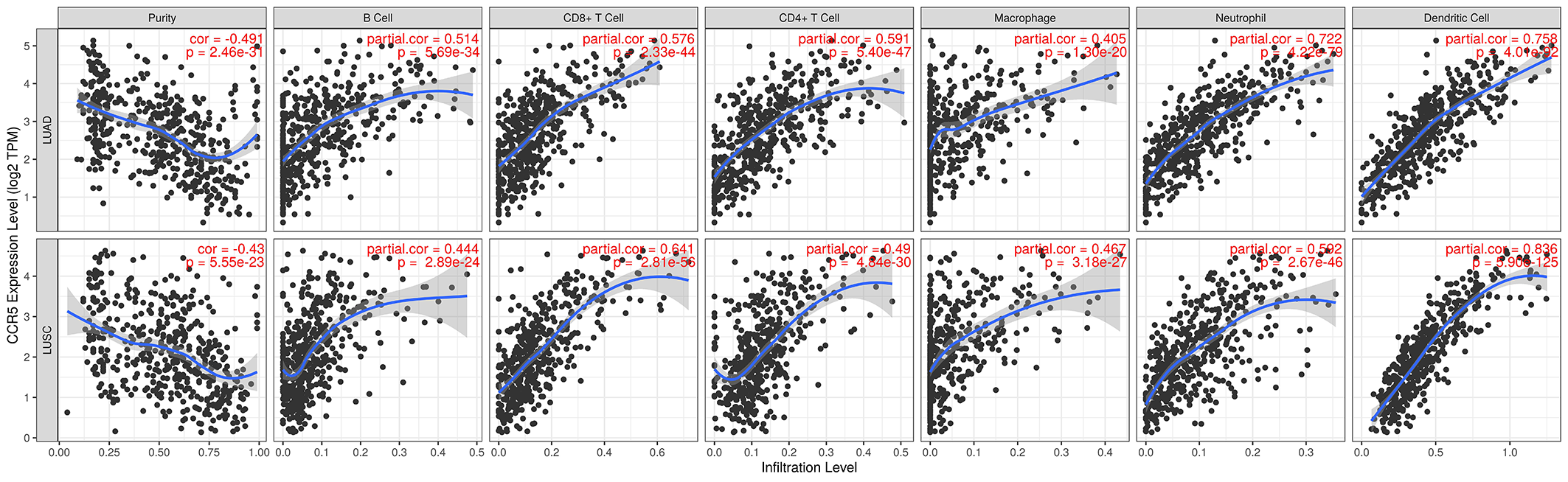

Supplement: Supplementary file 17 [file Image8.png]

# IL7R (226218\_at)

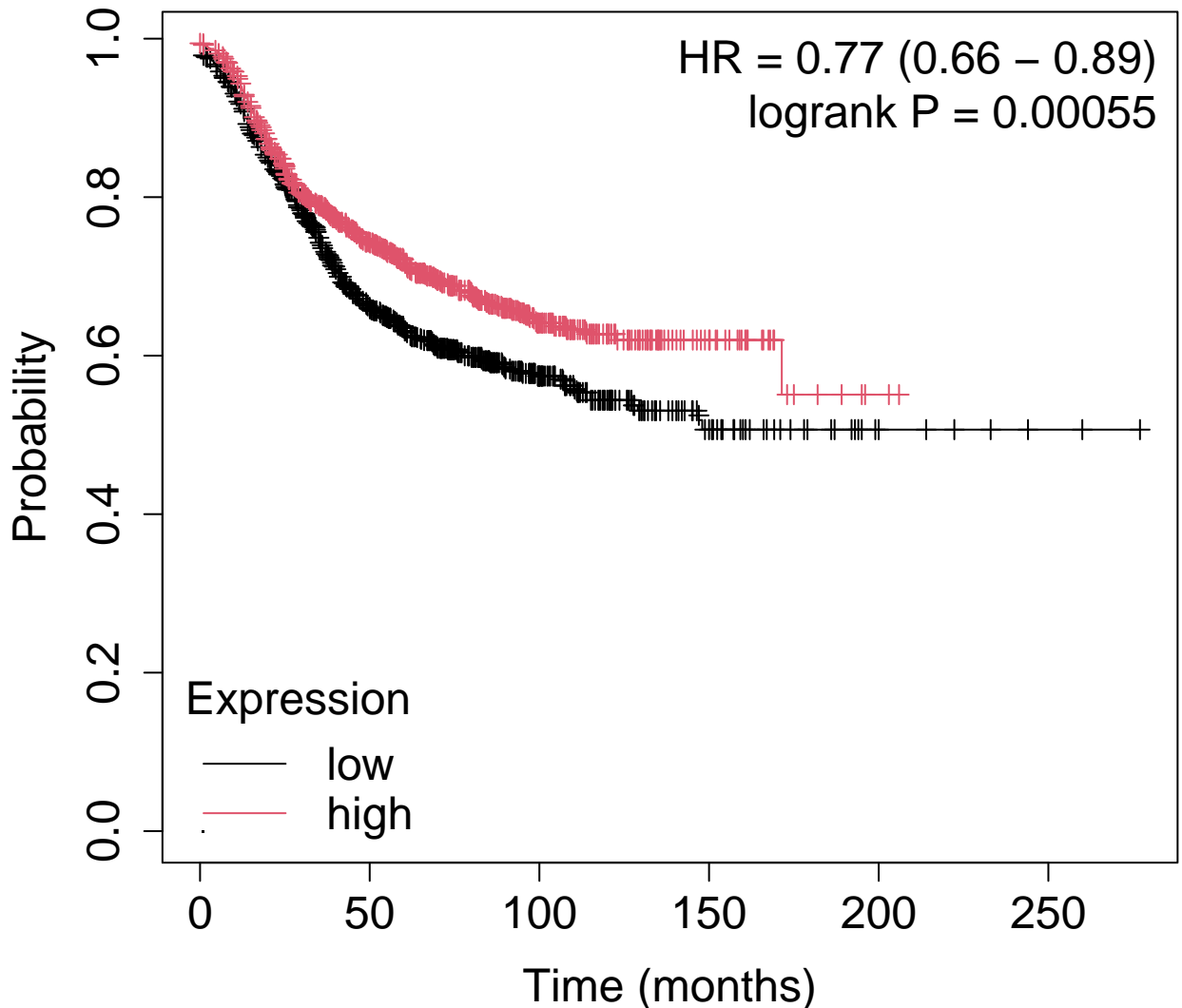

Number at risk

|      |      |     |     |    |   |   |
|------|------|-----|-----|----|---|---|
| low  | 1017 | 514 | 169 | 41 | 8 | 2 |
| high | 1015 | 578 | 177 | 27 | 2 | 0 |

Supplement: Supplementary file 18 [file DataSheet8.pdf]

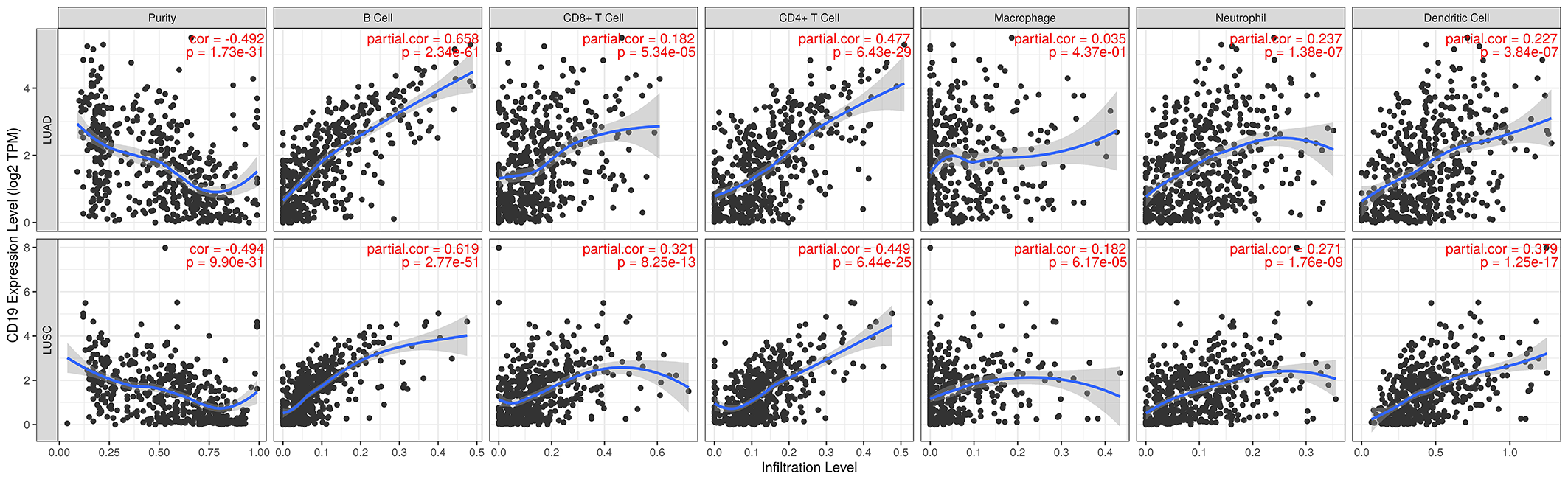

Supplement: Supplementary file 19 [file Image9.png]

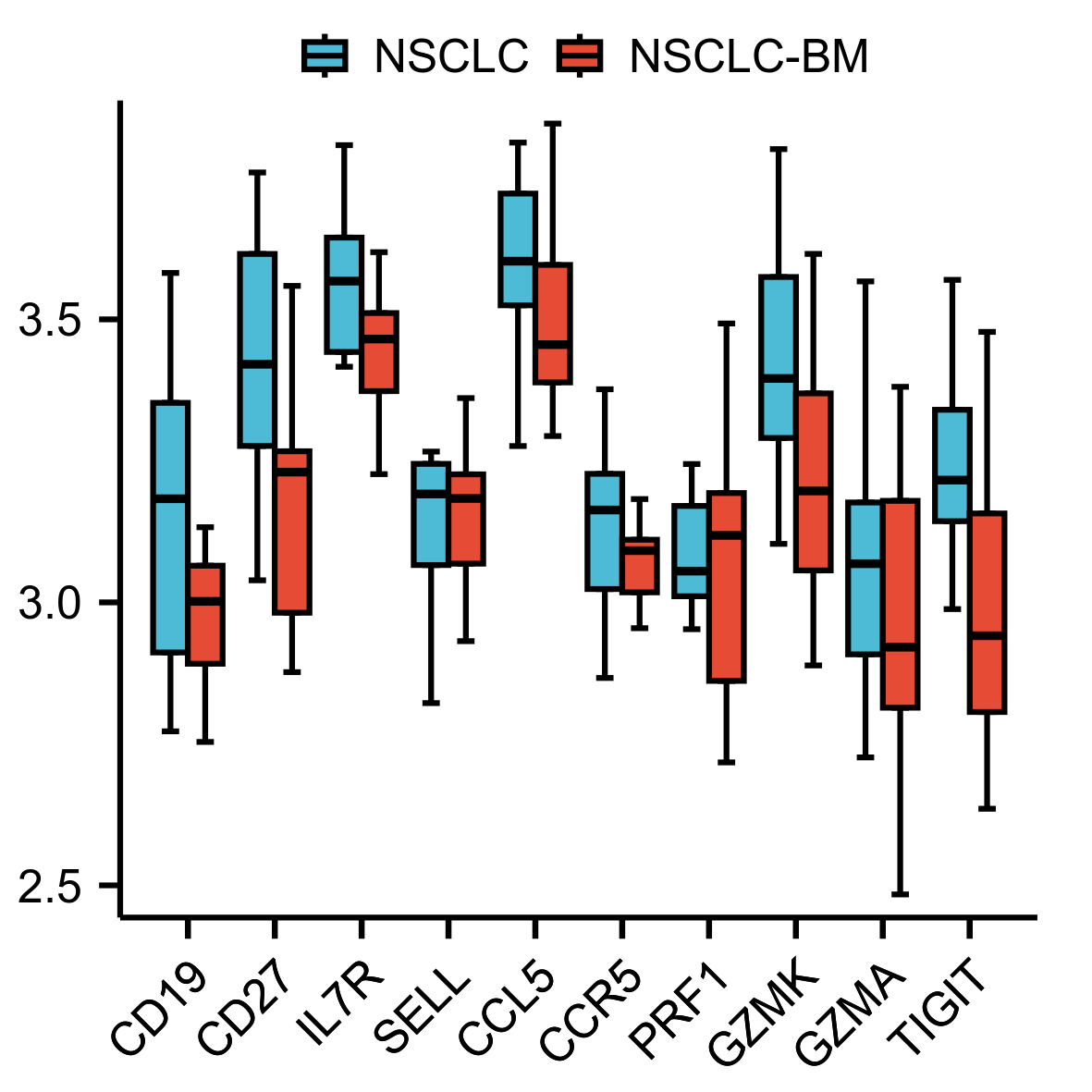

Supplement: Supplementary file 20 [file Image4.tiff]

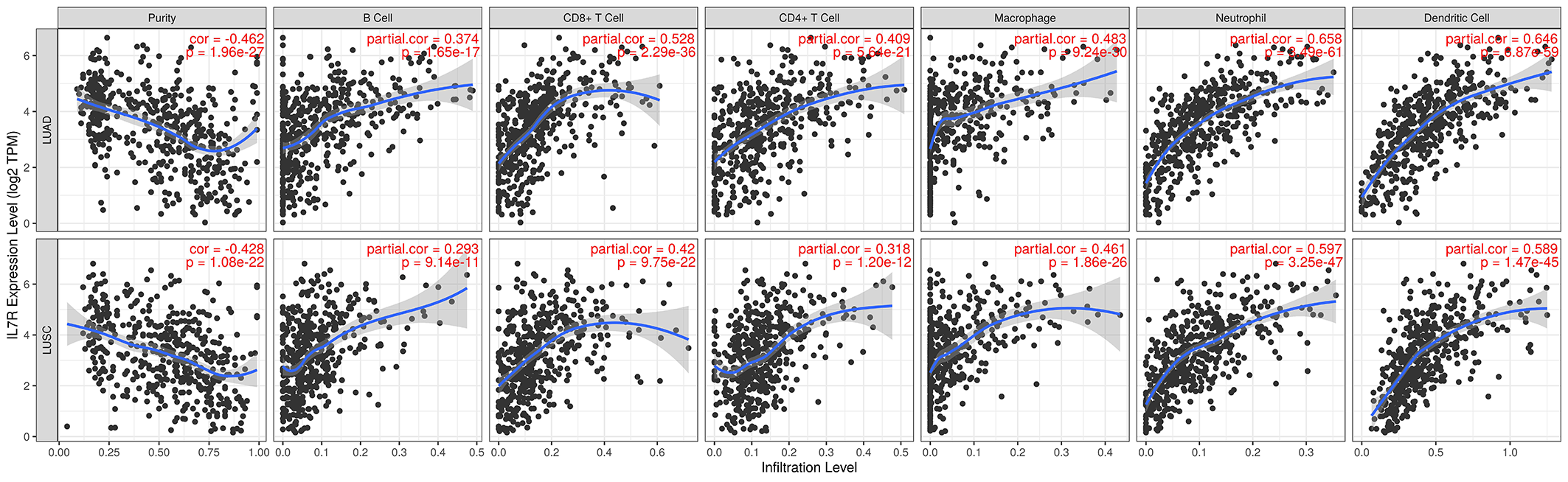

Supplement: Supplementary file 21 [file Image6.png]

# LSEL (204563\_at)

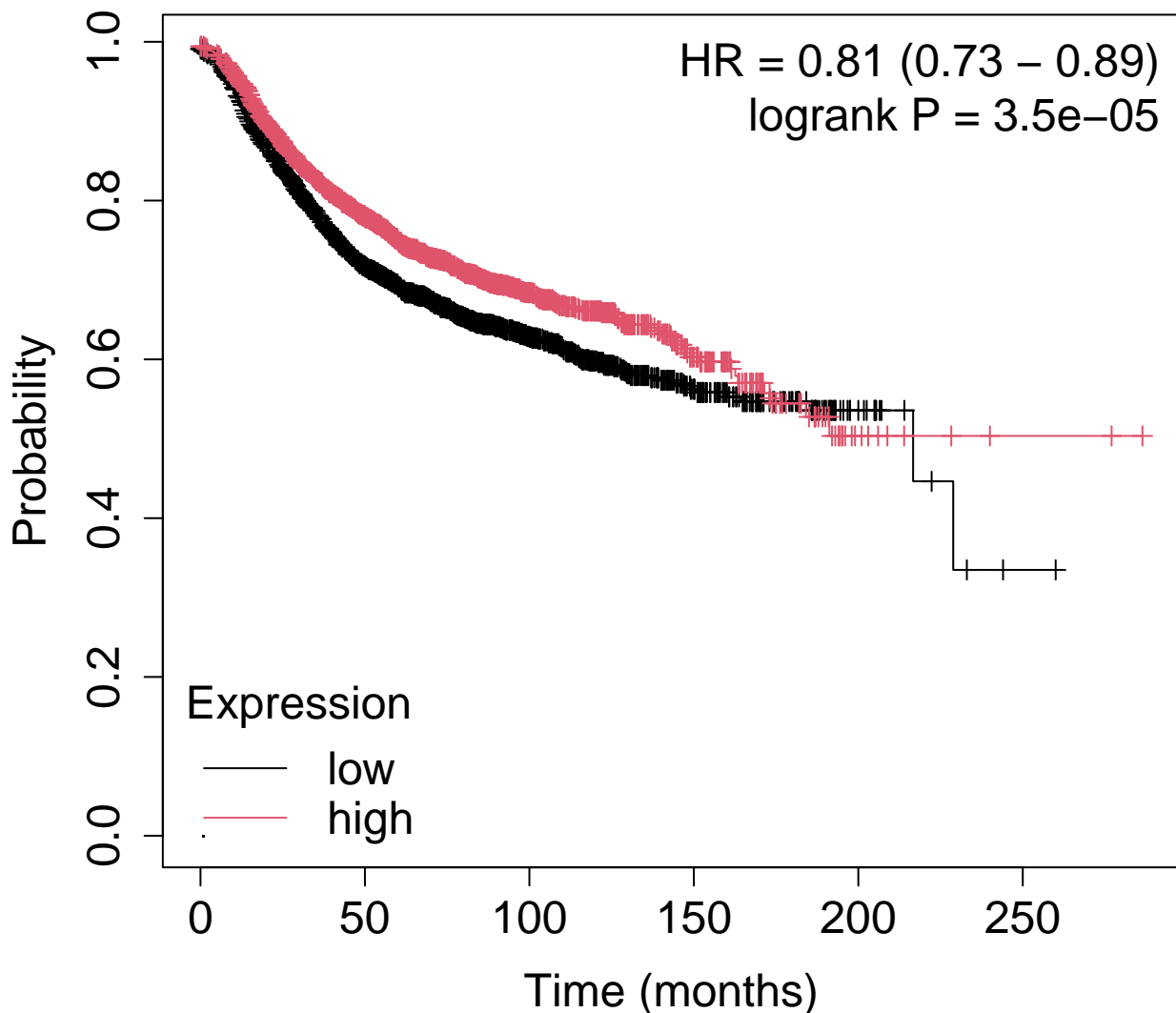

Number at risk

|      |      |      |     |     |    |   |
|------|------|------|-----|-----|----|---|
| low  | 2469 | 1443 | 587 | 139 | 17 | 1 |
| high | 2460 | 1440 | 549 | 107 | 10 | 2 |

Supplement: Supplementary file 22 [file DataSheet10.pdf]

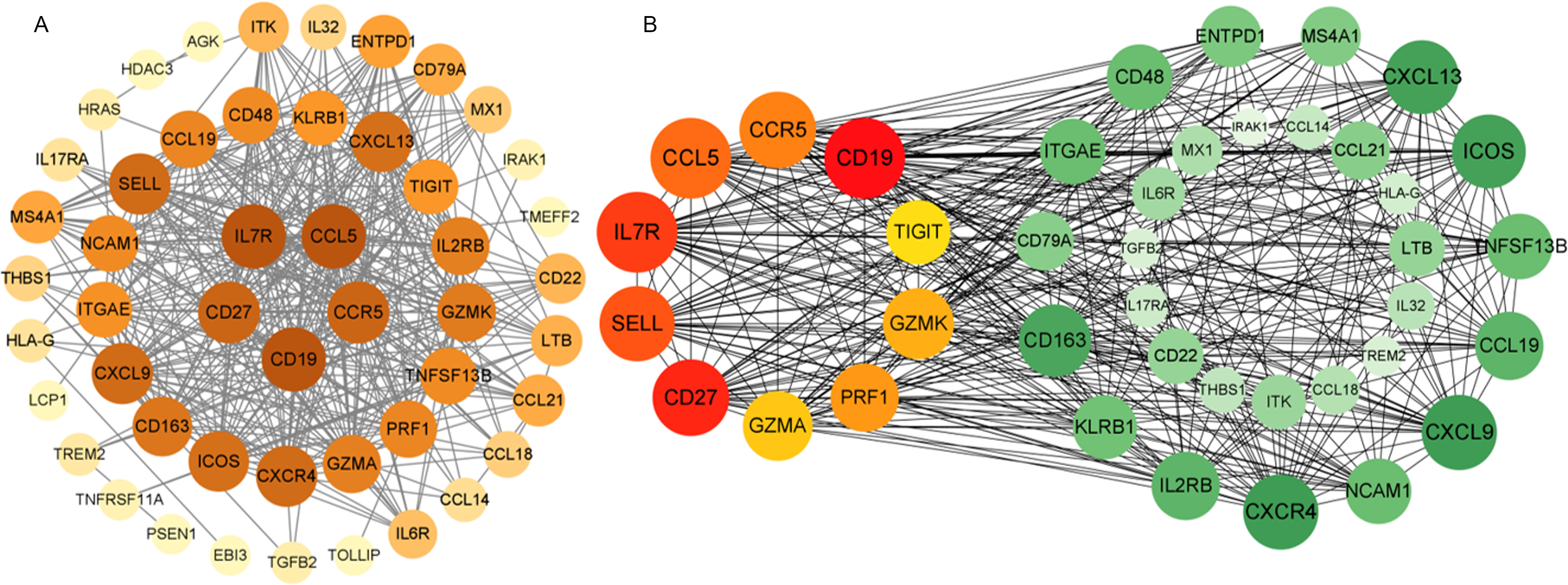

Supplement: Supplementary file 23 [file Image3.png]

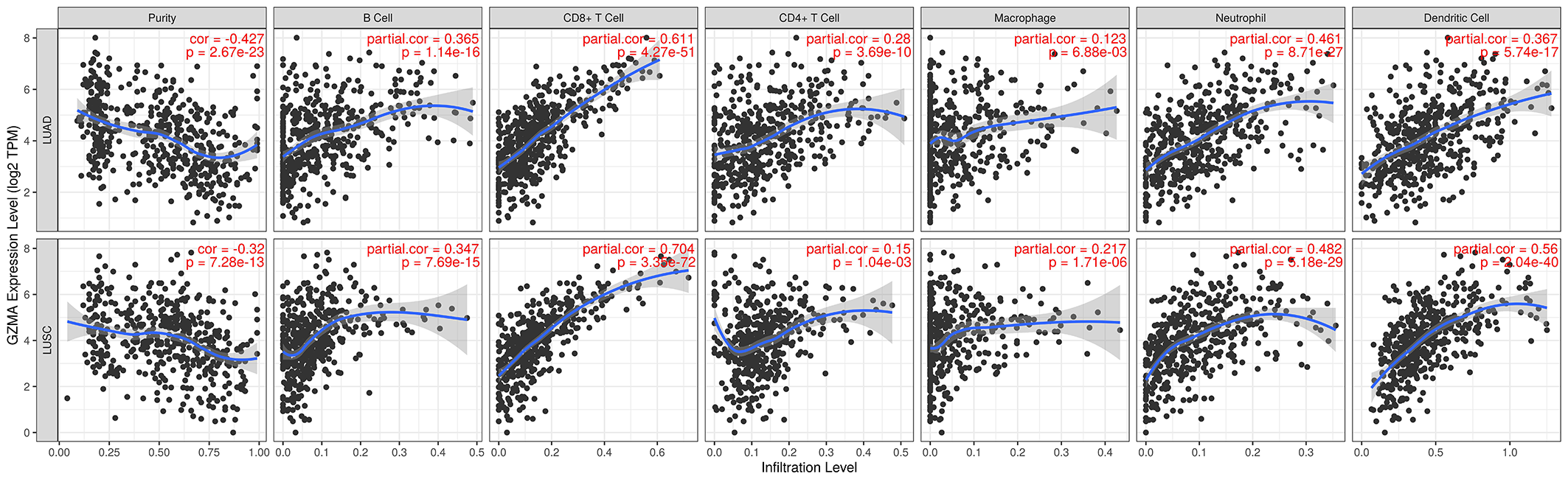

Supplement: Supplementary file 24 [file Image10.png]
